# Cyclopamine and Rapamycin Synergistically Inhibit mTOR Signalling in Mouse Hepatocytes, Revealing an Interaction of Hedgehog and mTor Signalling in the Liver

**DOI:** 10.3390/cells9081817

**Published:** 2020-07-31

**Authors:** Luise Spormann, Christiane Rennert, Erik Kolbe, Fritzi Ott, Carolin Lossius, Robert Lehmann, Rolf Gebhardt, Thomas Berg, Madlen Matz-Soja

**Affiliations:** 1 Rudolf-Schönheimer-Institute of Biochemistry, Faculty of Medicine, Leipzig University, Johannisallee 30, 04103 Leipzig, Germany; Christiane.Rennert2@medizin.uni-leipzig.de (C.R.); erikkolbe1@gmail.com (E.K.); Fritzi.Ott@medizin.uni-leipzig.de (F.O.); carolinlossius@outlook.de (C.L.); robert.lehmann@izi.fraunhofer.de (R.L.); Rolf.Gebhardt@medizin.uni-leipzig (R.G.); Madlen.Matz@medizin.uni-leipzig.de (M.M.-S.); 2 Department of Hepatobiliary Surgery and Visceral Transplantation, Leipzig University, Philipp-Rosenthal-Str. 55, 04103 Leipzig, Germany; 3 Division of Hepatology, Clinic and Polyclinic for Oncology, Gastroenterology, Hepatology, Infectious Diseases, and Pneumology, University Clinic Leipzig, Liebigstr. 19, 04103 Leipzig, Germany; thomas.berg@medizin.uni-leipzig.de

**Keywords:** mTOR, hedgehog, liver, hepatocytes, cyclopamine, rapamycin, mTORC2

## Abstract

In the liver, energy homeostasis is mainly regulated by mechanistic target of rapamycin (mTOR) signalling, which influences relevant metabolic pathways, including lipid metabolism. However, the Hedgehog (Hh) pathway is one of the newly identified drivers of hepatic lipid metabolism. Although the link between mTOR and Hh signalling was previously demonstrated in cancer development and progression, knowledge of their molecular crosstalk in healthy liver is lacking. To close this information gap, we used a transgenic mouse model, which allows hepatocyte-specific deletion of the Hh pathway, and in vitro studies to reveal interactions between Hh and mTOR signalling. The study was conducted in male and female mice to investigate sexual differences in the crosstalk of these signalling pathways. Our results reveal that the conditional Hh knockout reduces mitochondrial adenosine triphosphate (ATP) production in primary hepatocytes from female mice and inhibits autophagy in hepatocytes from both sexes. Furthermore, in vitro studies show a synergistic effect of cyclopamine and rapamycin on the inhibition of mTor signalling and oxidative respiration in primary hepatocytes from male and female C57BL/6N mice. Overall, our results demonstrate that the impairment of Hh signalling influences mTOR signalling and therefore represses oxidative phosphorylation and autophagy.

## 1. Introduction

The mechanistic target of rapamycin (mTOR) pathway has manifold functions in the liver involving both the regulation of metabolic activities in healthy organs and processes in the progression of diseases and tissue regeneration. To perform all these functions, the serine/threonine protein kinase mTOR forms multi-protein complexes termed mTOR complex 1 (mTORC1) and mTOR complex 2 (mTORC2), which have different compositions and responses to upstream signals [1]. In the liver, mTORC1, which consists of mTOR, mammalian lethal with Sec13 protein 8 (mLST8), Dishevelled, Egl-10 and Pleckstrin domain-containing mTOR-interacting protein (Deptor), regulatory-associated protein of mTOR (Raptor) and proline-rich protein kinase B (Akt) substrate (Pras40), is critical for controlling metabolic processes. Therefore, the main role for mTORC1 is the regulation of the energy status, which it mediates by promoting lipogenesis and lysosome biogenesis as well as inhibiting lipophagy and autophagy in response to growth factors, amino acids and oxygen levels [2]. In this process, mTORC1 regulates mitochondrial oxidative metabolism by initiating the translation of important mitochondrial genes located in the nucleus via Eukaryotic translation initiation factor 4E-binding protein 1 (4E-BP1) downstream signalling [3,4]. However, Ribosomal protein S6 kinase beta-1 (p70S6)-induced downstream signalling can also influence mitochondrial ATP production and energy metabolism [5]. Similar to mTORC1, mTORC2 consists of mTOR, mLST8 and Deptor, and it also contains rapamycin-insensitive companion of mTOR (RICTOR) and mammalian stress-activated protein kinase-interacting protein (mSIN1) [2]. In contrast to mTORC1, mTORC2 is responsible for cell survival and migration, and it is closely associated with the insulin/phosphoinositide 3-kinase (PI3K) pathway and Akt signalling pathway [1]. In this regard, recent studies have shown that mice in which mTORC2 is downregulated in the liver (via deletion of RICTOR) cannot sense satiety while in a fed state because of enhanced gluconeogenesis and inhibited glycolysis, glycogen synthesis, and lipogenesis [6]. In accordance with their different agonists in vivo, both complexes have different sensitivities to inhibitors, such as rapamycin, which mainly inhibits mTORC1 downstream signalling via p70S6 [7], whereas TORIN supresses both mTORC1 and mTORC2 signalling [8].

Although the molecular mechanisms driving the regulation of metabolic processes via mTOR signalling are well understood in principle, there are still major gaps in the research on the crosstalk of mTOR with signalling pathways known to serve as gatekeepers for lipid metabolism in the liver. Recently, we discovered that the Hedgehog (Hh) pathway is an essential player in liver lipid metabolism and metabolic zonation, influencing circadian rhythms [9,10,11]. The Hh pathway can be induced by binding of the Hh ligands Indian Hh, Sonic Hh and Dessert Hh to the receptors Patched (PTCH). Ligand binding activates the co-receptor Smoothened (SMO), which results in the release of the full-length glioma-associated oncogene family zinc finger (GLI) transcription factors 1–3 from the suppressor of fused (SUFU), with which it had been sequestered. Finally, the GLI transcription factors are translocated to the nucleus to activate Hh-specific target genes [12]. 

With respect to the crosstalk of the mTOR and the Hh pathway, oncological contexts, such as basal cell carcinoma and gastrointestinal cancer, including oesophageal cancers, are particularly well known and described [13,14]. However, the conditions in tumours characterized by a strong activation of the Hh and/or mTOR signalling cascades are completely different than those in healthy tissue. To date, no investigations have been carried out to determine the crosstalk between the Hh and mTOR pathways in healthy liver and to indicate the ways they may co-modulate aspects of hepatic metabolism. However, since we consider it immensely important to understand the interactions of the networks formed by these highly active metabolic pathways, we conducted this study. Since energy metabolism is known to be affected by liver sexual dimorphism, we also wanted to know whether the crosstalk differs in males and females. By using mice in which hepatocyte-specific Hh has been deleted and in vitro experiments, we demonstrate a strong mutual interaction between Hh signalling and the mTOR pathway in healthy mouse hepatocytes. This study reveals new insights on the high complexity of the signalling network that controls liver energy metabolism and contributes to our comprehension of diseases caused by the dysregulation of these two pathways like non-alcoholic fatty liver disease or macrocephaly-associated conditions [15,16,17]. Therapies often target only one signalling pathway; however, targeting several parts of the regulating network may be a substantially more effective approach.

## 2. Materials and Methods 

### 2.1. Maintenance of the Mice and Feeding

Mice were maintained according to the German guidelines and local regulations for the care and safe use of experimental animals in a pathogen-free facility in a 12:12 h light-dark cycle (permission numbers: TVV44/16; T04/14). The animals were fed *ad libitum* with regular chow (ssniff^®^ M-Z V1124-0 composed of 22.0% protein, 50.1% carbohydrate, 4.5% fat; usable energy: 13.7 kJ/g; ssniff^®^ Spezialdiäten GmbH, Soest, Germany) and tap water throughout life. The mice were sacrificed at 3 months of age between 8 and 11 am after administration of an anaesthetic consisting of ketamine, xylazine and atropine. 

### 2.2. SAC Mice

The characteristics and the phenotype of the SAC mice have been previously described in Matz-Soja et al. 2014 [18].

### 2.3. Isolation and Cultivation of Primary Mouse Hepatocytes

The primary hepatocytes were isolated from male and female transgenic and C57Bl/6N mice using a collagenase perfusion technique as described before [19,20]. All hepatocytes from C57BL/6N mice were additionally cleared of non-parenchymal hepatocytes by differential centrifugation [18]. Afterwards, the hepatocytes were suspended in Williams Medium E containing 10% fetal calf serum, 20 µM glutamine, penicillin and streptomycin, and 90 pM dexamethasone and seeded in cell culture plates coated with collagen type I. After 2 to 3 h the medium was changed to serum-free media, which was used during further cultivation. 

### 2.4. Treatment with Cyclopamine, Rapamycin and Torin

For the treatment of the primary hepatocytes from C57BL/6N mice with the inhibitors, we used cells from one mice and treated them with final concentrations of 10 µM cyclopamine (LC Laboratories, Woburn, MA, USA), 50 nM rapamycin (Sigma-Aldrich, St. Louis, MO, USA) or 5 µM Torin (Axon Medchem LLC, Reston, VA, USA) diluted in dimethyl sulfoxide (DMSO) (Carl Roth GmbH + Co. KG, Karlsruhe, Germany) were added to the serum-free media. For combinatory treatment with cyclopamine, higher concentrated inhibitors were added to reach the final concentration with the same amount of DMSO. As controls we used media without any addition, to detect possible effects of the DMSO, and media added with the same amount of DMSO as was added with the inhibitors. This experiment was repeated 3–5 times with different male and female mice.

### 2.5. Sample Preparation for Proteome Profiling

Hepatocytes were lysed at 95 °C for 10 min in lysis buffer containing 1% sodium deoxycholate (SDC), 10 mM tris(2-carboxyethyl)phosphine (TCEP), 40 mM chloroacetamide (CAA), and 100 mM Tris pH 8.5. Then, the hepatocytes were sonicated for 5 min. The digestion and purification of the lysates were conducted using the PreOmics GmbH (Martinsried, Germany) in-Stage Tip kit (iST kit 96×). Next, the samples were sequentially eluted into three fractions using the SDB-RPS-1 and SDB-RPS-2 buffers [21] and the elution buffer provided by PreOmics. The subsequent analysis was carried out with a nanoLC-MS system.

### 2.6. LC-MS Instrument Settings for Shotgun Proteome Profiling and Data Analysis

LC−MS/MS was performed using nanoflow reverse-phase liquid chromatography (Dionex Ultimate 3000, Thermo Fisher Scientific Inc., Waltham, MA, USA) coupled online to a Q Exactive HF Orbitrap mass spectrometer (Thermo Fisher Scientific Inc.). The LC separation was conducted using a PicoFrit analytical column 75 μm ID × 55 cm long, 15 µm Tip ID (New Objective, Inc., Woburn, MA, USA) that was packed in-house with 3 µm C18 resin (ReproSil-AQ-Pur, Dr. Maisch GmbH, Ammerbuch-Entringen, Germany), as reported previously [22]. Briefly, peptides were eluted on a gradient from 3.8 to 50% solvent B in solvent A for 121 min at a 266 nL per minute flow rate. Solvent A consisted of 0.1% formic acid and solvent B was 79.9% acetonitrile, 20% water and 0.1% formic acid. Nanoelectrospray ionization was achieved at 3.5 kV. A cycle of one full Fourier transformation scan mass spectrum (300−1750 *m*/*z*, resolution of 60,000 at *m*/*z* 200, AGC target 1 × 10^6^) was followed by 12 data-dependent MS/MS scans (resolution of 30,000, AGC target 5 × 10^5^) with a normalized collision energy of 25 eV. To avoid repeatedly sequencing the same peptides a dynamic exclusion window of 30 s was used. Furthermore, only peptide charge states between two and eight were sequenced.

MaxQuant software (1.5.7.4) [23] was used to analyse the raw MS data with the Andromeda search engine [24] and the mouse UniProtKB database with 51,416 entries released in 03/2016. A minimum peptide length of 7 amino acids, a false discovery rate (FDR) of 0.01 for proteins and peptides, a first search mass tolerance for peptides of 20 ppm and a main search tolerance of 4.5 ppm were the set criteria. For the tryptic digest, a maximum of two missed cleavages was allowed. Cysteine carbamidomethylation was set as the fixed modification, while N-terminal acetylation and methionine oxidation were set as variable modifications. Up- and downregulated proteins in the transgenic SAC-knockout (KO) mice were laid over a global molecular network developed from information contained in the Knowledge Base of the Ingenuity Pathway Analysis (IPA) software from Qiagen (Venlo, The Netherlands). The networks of these genes of interest were then algorithmically generated based on their interrelationships.

### 2.7. RNA Isolation and Quantitative Real-Time PCR (qPCR)

Total RNA was isolated using peqGOLD RNAPure™ (VWR International, Radnor, PA, USA). Reverse transcription of the RNA and the cDNA synthesis was performed using a ProtoScript^®^ First Strand cDNA synthesis kit (New England Biolabs, Ipswich, MA, USA) according to the manufacturer’s instructions. The quantity of the cDNA was measured in duplicates with qPCR with a gene-specific and intron-spanning primer designed with Primer 3 software for every target gene. A Rotor-Gene SYBR^®^ Green PCR kit and a Rotor-Gene Q (Qiagen) were used according to the manufacturer’s instructions. The amount of specific PCR products for each primer set was quantified by applying the standard curve method. *β-Actin* (*Actb*) was amplified in all samples of SAC mice and *14-3-3 protein zeta/delta* (*Ywhaz*) for all samples from the C57BL/6N mice for normalization as the reference gene. Primer sequences can be found in Table 1.

### 2.8. ATP-Assay

The ATP content was determined as described in Matz-Soja et al. 2016 [10]. 

### 2.9. Seahorse Analysis

Isolated hepatocytes were seeded on collagen-coated XF96 cell culture microplates (Agilent Technologies, Inc., Santa Clara, CA, USA) (1.5 × 10^4^ cells/well). Mito Stress Test medium was prepared from a DMEM base, which was supplemented with 11 mM glucose, 0.23 mM sodium pyruvate, and 2 mM glutamine (final concentrations). The Mito Stress Test was performed according to the manufacturer’s instructions using the final concentrations of 2 µM oligomycin and 0.25 µM carbonyl cyanide-4-(trifluoromethoxy)phenylhydrazone (FCCP). The raw data was analysed using Wave software (Agilent Technologies, Inc.). The energy phenotype analysis was based on the measurements obtained from the Mito Stress Test.

### 2.10. Akt Signalling Phosphorylation Array

To analyse the phosphorylation state of the Akt pathway we used the Human/Mouse AKT Pathway Phosphorylation Array C1 from RayBiotech (Peachtree Corners, GA, USA). Primary hepatocytes from male SAC mice were isolated and stored at −80 °C. Subsequently, they were lysed and analysed according to the manufacturer’s instructions. 

### 2.11. Image Detection and Densitometric Analysis

The Western blot and chemiluminescent images were captured with an Odyssey^®^ Fc Imaging System from LI-COR Biosciences (Lincoln, NE, USA) and the densitometric analyses were performed with the Image Studio™ from LI-COR Biosciences.

### 2.12. Flow Cytometry

Hepatocytes were isolated and suspended in culture medium. For MitoTracker staining, the hepatocytes were incubated with 100 nM MitoTracker™ Green FM (Thermo Fisher Scientific) for 15 min at 37 °C. Then the hepatocytes were switched to fresh culture medium. For JC-10 staining, the hepatocytes were incubated with JC-10 for 30 min at 37 °C according to the manufacturer’s instructions. Hepatocytes, which have been pre-incubated with 100 µM FCCP for 10 min served as the negative control for the membrane potential measurements. The results from both staining experiments were analysed with a BD LSR II flow cytometer (Becton Dickinson GmbH, Heidelberg, Germany).

### 2.13. Western Blotting

After isolation, the hepatocytes were lysed in RIPA buffer and homogenized using Precellys^®^ 24 homogenizer (VWR International). Protein concentration was determined using BCA assay (Thermo Fisher Scientific Inc.) according to the manufacturer’s instructions. A total of 15–20 µg of protein was separated using 6–20% SDS-PAGE (Bio-Rad Laboratories, Inc., Hercules, CA, USA). A tank blot system (Bio-Rad Laboratories, Inc.) was used for immunoblotting the proteins to a nitrocellulose membrane (LI-COR Biosciences). The membranes were blocked with Odyssey^®^ blocking buffer in TBS (#927-60001) (LI-COR Biosciences) and incubated with primary antibodies against mTOR (#2972), pmTOR (Ser2448) (#2971), Raptor (#2280), pRaptor (Ser792) (#2083), Pras40 (#64815), pPras40 (Thr246) (#64815), Rictor (#9476), pRictor (Thr1135) (#3806), p70S6 (#2708), pp70S6 (Thr389) (#9234), 4E-BP1 (#9644) and p4E-BP1 (Thr37/46) (#2855) (Cell Signaling Technology, Inc., Danvers, MA, USA). All primary antibodies were diluted 1:1000 and the secondary antibody was diluted 1:10,000 in blocking buffer. The secondary antibody IRDye^®^ 800CW goat anti-rabbit IgG (#926-32211) (LI-COR Biosciences) and the Odyssey^®^ Fc imager (LI-COR Biosciences) were used for the detection and quantification of the proteins. The total amount of the applied protein was determined using REVERT™ Total Protein Stain (LI-COR Biosciences) and used for normalization. 

### 2.14. Sample-Size Estimation

When the study was being designed, the appropriate sample size was determined using our experience from previous studies with liver-related experiments with the transgenic and the non-transgenic mice [10,18,25]. For the isolated hepatocytes from the transgenic SAC-wildtype (WT), SAC-KO, and C57BL/6N mice, the same procedure was used. 

### 2.15. Study Design and Statistical Analyses

The number of biological replicates varies by experiment (*n* = 3–7) as specified in each figure. Depending on the type of experiment, the number of technical replicates also varied, but most experiments were performed in duplicate or triplicate. The identification of the outliers was conducted using a ROUT test with the software GraphPad Prism 7 (GraphPad Software, San Diego, CA, USA) (aggressiveness 1%). The following statistical analyses and the data depicted in the figures were prepared with the cleaned data. The values are presented as the means of the biological replicates ± standard error of the mean. The statistical evaluation for C57BL/6N mice was performed with one-way ANOVA for repeated measurements (GraphPad Prism 7). For the statistical analysis of the SAC mice, multiple t-tests were used (GraphPad Prism 7). P-values are indicated as *p* < 0.05 (*), *p* < 0.01 (**) and *p* < 0.001 (***) levels. We referred to values 0.05 > *p* > 0.005 as suggestive evidence for significance and *p* < 0.005 as significant values. 

## 3. Results

### 3.1. Hh Signalling Influences the Energy Phenotype of Murine Hepatocytes by Targeting Mitochondrial Function

**Figure 1:**
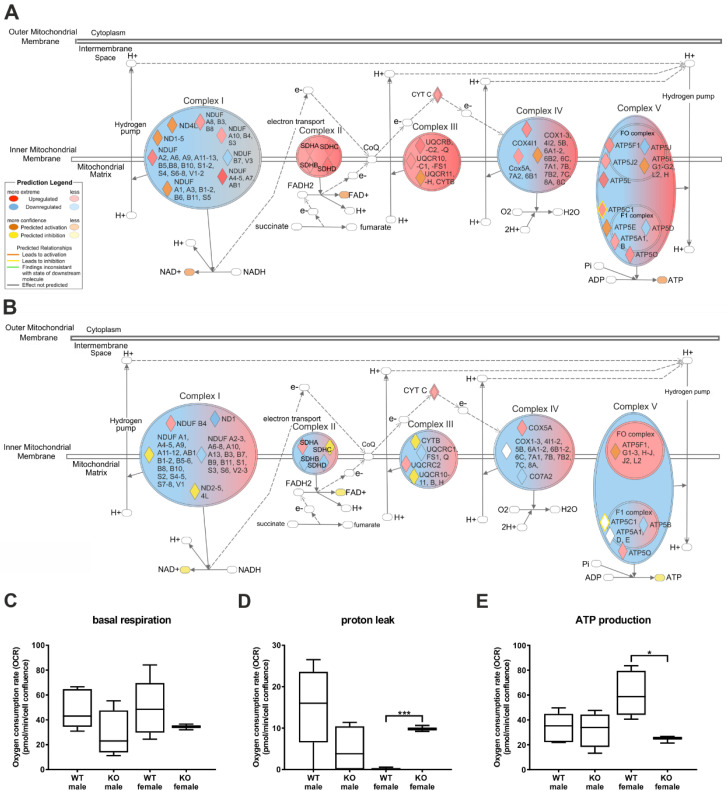
The impact of the hepatocyte-specific knockout of Hedgehog signalling on mitochondrial respiration. (**A**,**B**): Ingenuity Pathway analysis (IPA) of the electron transport chain based on proteome data of male (**A**) and female (**B**) SAC mice. (**C**–**E**): Seahorse analysis Mito Stress Test of SAC mice. Data are normalized to the respective wildtype (WT). Significance was calculated with unpaired t-test. Stars show the significance to WT animals. * *p* ≤ 0.05, *** *p* ≤ 0.001; *n* = 3−8.

To get a first impression of the impact of Hh signalling alterations on the energy phenotype of the hepatocytes, we used isolated hepatocytes from transgenic male and female mice in which *Smo* was conditionally deleted (SAC mice) and performed a Seahorse Energy Phenotype assay. The results of this analysis indicated that the hepatocytes from the male SAC-knockout (KO) mice showed a slight shift to a glycolytic phenotype, whereas the female SAC-KO mice exhibited a slight shift to a quiescent phenotype (Figure S1A,B). To investigate this result in greater detail, we performed a proteome analysis of those hepatocytes. Subsequently, we used Ingenuity Pathway Analysis (IPA) software, which compared to gene-set-enrichment methods, additionally applies literature-based cause–effect relationships [26]. With this software we compute predictions of the behaviour of all the metabolic processes based on the abundance of individual proteins. One of the striking results of this study was the strong impact of the Hh changes on mitochondrial function via alterations in the electron transport chain. Specifically, the male SAC-KO mice exhibited downregulation of complex I in the electron transport chain as indicated by an increase in the NADH dehydrogenase 1 alpha subcomplex subunit 2 (NDUFA2), NDUFA4–13, AB1, B3–5, B8, B10, S1–4, S6–8 and V1–2 levels, whereas the NDUF B7 and V3 levels were decreased (Figure 1A), which led to a predicted increase in NAD^+^. In contrast, complexes II and III, as well as cytochrome C were upregulated, as indicated by elevated succinate dehydrogenase flavoprotein subunit (SDHA) SDHB-D and Cytochrome b-c1 complex subunit 7 (UQCRB), UQCRC2 and UQCRQ, as well as the UQCR10C1 and Bcl-2-related protein A1 selective peptide (FS1) levels, which subsequently led to an increase in FAD^+^. In complex IV, the subunits Prostaglandin G/H synthase (COX) 4I1, 5A, 7A2 and 6B1 were upregulated; nevertheless, the IPA software predicted only partial upregulation of the whole complex. Similar results were obtained for complex V of the electron transport chain (the ATP synthase). Specifically, the levels of the adenosine triphosphate synthase subunit alpha 1 (ATP5A1), ATP5B, C1, F1, J2, L and O proteins were increased, while the ATP5 D and J levels were reduced. With these extensive alterations, the IPA software predicted an increase in adenosine triphosphate (ATP) in the male SAC-KO mice. In contrast to the male mice, the alterations in protein expression in regard to Hh signalling in the female SAC-KO mice were not predicted to lead to a clear trend of up- or downregulation for complexes I-IV (Figure 1B). For example, in complex I, only the NDUF B4 level was elevated in the female SAC-KO mice similar to that in the SAC-KO males, however the ND1, NDUF A2–3, A6–8, A10, A13, B3, B7, B9, B11, S1, S3, S6 and V2–3 levels were decreased in the SAC-KO females. In complex II, the level of subunit SDHA was increased in the female mice with abolished hepatic Hh signalling similar to the trend in the male SAC-KO mice. In contrast, the SDHB and D levels were reduced in the SAC-KO females. In complex III in the female SAC-KO mice, only the level of subunit UQCRC2 was increased similar to that of the SAC-KO males, whereas the UQCRC1, FS1 and Q levels were reduced. In addition, the COX5A level was increased in the female SAC-KO mice and the COX7A2 level was reduced. Nevertheless, the level of cytochrome C was increased in both the female and male SAC-KO mice. However, in contrast to the male mice with hepatocyte-specific deletion of the Hh pathway, IPA predicted the downregulation of NAD^+^ and FAD^+^ in the female SAC-KO mice. Interestingly, complex V in the female SAC-KO mice was downregulated with elevated ATP5O and reduced ATP5B levels, causing a predicted decrease in the amount of ATP (Table S1 and S2). 

To confirm the results of the proteome data analysis, we investigated the functionality of the mitochondrial electron transport chain through measurements of the basal oxygen consumption rate (OCR) and the basal extracellular acidification rate (ECAR). We detected no changes in the basal respiration for either the male or female mice (Figure 1C). Next, we measured OCR and ECAR after blocking ATP synthase to assess the amount of oxygen used for ATP production. The results showed a significant increase in the proton leak and suggestive evidence for a significant decrease in ATP production in female SAC-KO mice, compared to the female WT mice (Figure 1D,E). In contrast to the hepatocytes from the female mice, hepatocytes from male KO mice showed a non-significant decrease in the proton leak and no alteration in the ATP production compared to male WT hepatocytes. Additionally, we uncoupled the proton gradient to determine the maximal respiratory capacity of the hepatocytes, which was not different in the male and female SAC-KO hepatocytes (Figure S1C,D). Finally, we blocked complex I and III to determine the amount of oxygen consumed by the hepatocytes for other processes and detected no alterations in the mice of either sex (Figure S1E).

Because of the changes in mitochondrial respiration, we looked at the gene expression of key regulators of mitochondrial function. In the male SAC-KO mice we detected suggestive evidence for a significant increase compared to male SAC-WT mice in solute carrier family 25 member 1 (*Slc25a1*) and *Slc25a20*, which translate transporter proteins that support the maintenance of the citric acid cycle and a significant increase in *Slc25a47*, which encodes an uncoupling protein (Figure S1F). None of the three genes were affected in the hepatocytes from the female SAC-KO mice (Figure S1G). In contrast, compared to the respective WT mice, *Slc25a4*, which encodes an ADP/ATP carrier protein, was not affected in the hepatocytes from the male SAC-KO mice, but its expression was elevated in the female SAC-KO mice. The mitochondrial ornithine transporter *Slc25a15* did not appear to be altered in the mice of either sex. 

**Figure 2:**
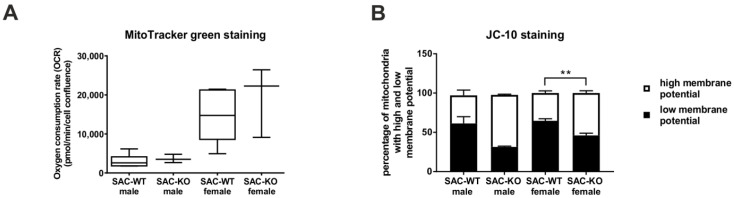
Effect of hepatocyte-specific Hedgehog knockout on mitochondrial mass and membrane potential. (**A**): Mitochondrial mass of SAC mice. (**B**): mitochondrial membrane potential. Error bars show the standard error of the mean (SEM). Significance was calculated with multiple t-test. Stars show the significance to WT animals. ** *p* ≤ 0.01; *n* = 2–7.

To determine whether these observed alterations were caused by a principal change in mitochondrial features, we analysed the mitochondrial mass and membrane potential using flow cytometry. The findings of these experiments showed that the deletion of Hh signalling had no impact on the mitochondrial mass in the mice of either sex (Figure 2A, Figure S2B). However, the results of the JC-10 staining experiments showed suggestive evidence for a significant increase in the mitochondrial membrane potential of female SAC-KO mice, whereas the male SAC-KO mice showed only a trend for this observation (Figure 2B, Figure S2C,D).

In summary, the hepatocyte-specific alteration of Hh signalling in vivo had a great impact on the mitochondrial function in hepatocytes. It led to hyperpolarized mitochondria and impairment of mitochondrial ATP production in the hepatocytes from the female mice. 

### 3.2. Hh Influences Mitochondrial Metabolism and Autophagy by Stimulating mTOR Signalling 

**Figure 3:**
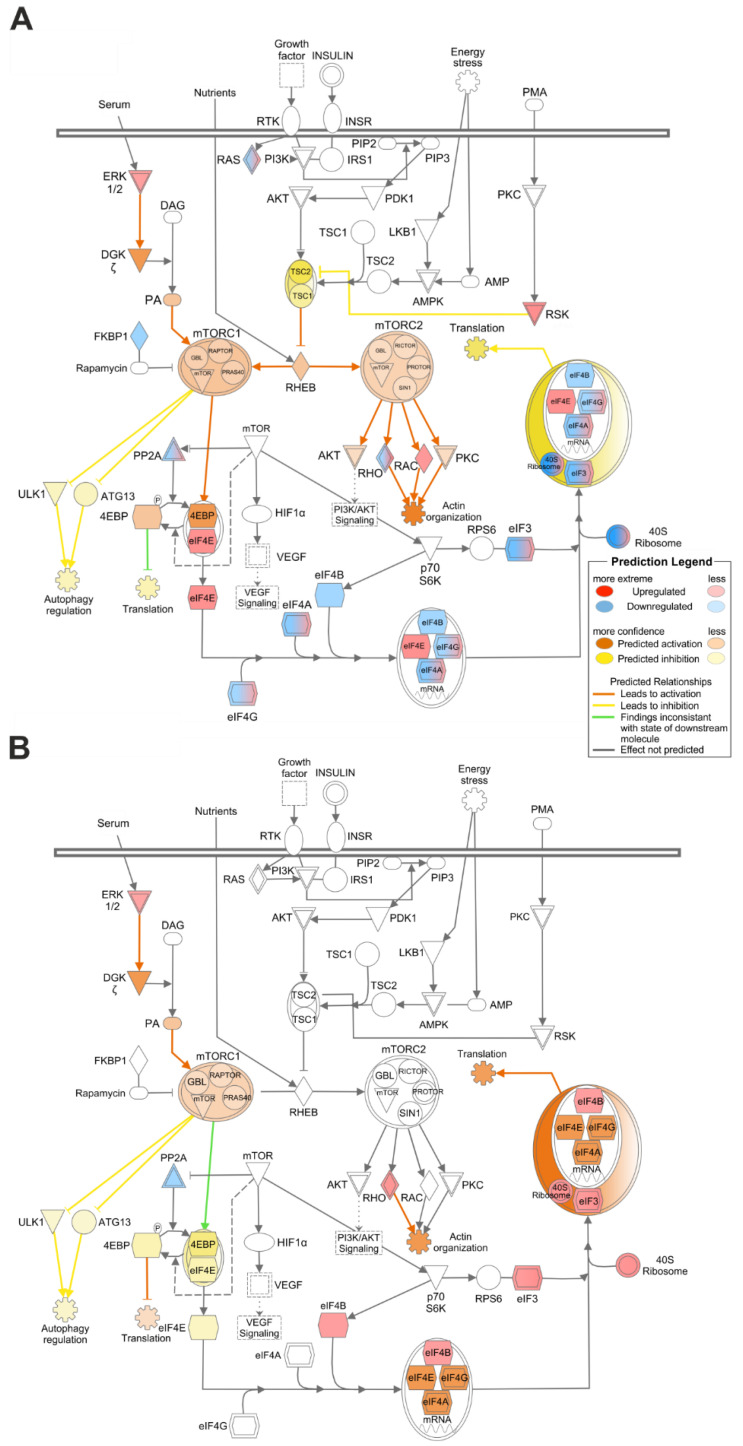
Impact of the hepatocyte-specific knockout of Hedgehog signalling on mechanistic target of rapamycin (mTOR) signalling. A-B: IPA analysis of mTOR signalling based on proteome data of male (**A**) and female (**B**) SAC mice.

Since energy metabolism in hepatocytes is strongly connected to mTOR signalling we analysed the proteomic study described above with respect to potential changes in mTOR signalling. The results showed the activation of the extracellular signal-regulated kinase 1/2 (ERK1/2) in the hepatocytes with specifically deleted Hh signalling in both sexes (Figure 3A,B). Therefore, the IPA software predicted the activation of the mTORC1 complex and the inhibition of autophagy in both sexes. In the male mice, the peptides form protein phosphatase 2 (PP2A), eukaryotic translation initiation factors (elFs) 3, 4A, 4G, and the 40S ribosome were partly down- and upregulated. Additionally, elF4E was upregulated and elF4B was downregulated, causing a predicted inhibition in translation. In contrast to the expression in the male SAC-KO mice, the female mice exhibited upregulated elF3 and 4B, and 40S ribosome, and subsequently, their translation was predicted to be upregulated. When we looked at the downstream signalling of the mTORC2 complex, RHO was mainly downregulated, but was also partly upregulated, whereas RAC was only upregulated. In contrast, RHO was upregulated in the female mice. However, the IPA software predicted activation of actin organization for both sexes (Tables S3 and S4). 

**Figure 4:**
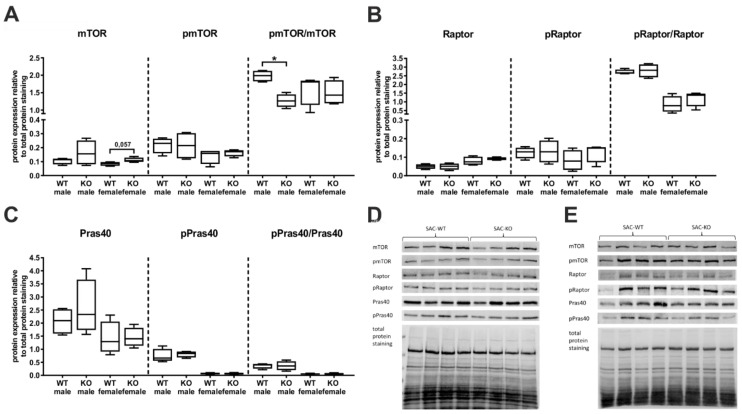
Western blot analysis of the impact of the hepatocyte-specific knockout of Hedgehog signalling on mechanistic target of rapamycin (mTOR) signalling. Densitometric analysis of the Western blots of (**A**): mTOR and its phosphorylation (p) at Ser2448. (**B**): Raptor and its phosphorylation (p) at Ser792. (**C**): Pras40 and its phosphorylation (p) at Thr246. (**D**,**E**): Representative example of the Western blots of male (**D**) and female (**E**) mice. * *p* ≤ 0.05; *n* = 4.

In addition to the prediction of the IPA software, we performed Western blot analysis, which showed only a small tendency of increased amount of mTOR in both sexes, whereby the results did not reach significant values. Besides, the quantity of mTOR phosphorylated at Ser2448 was also not altered (Figure 4A,D,E). Nevertheless, the ratio of the amount of phosphorylated mTOR to the total amount of mTOR showed suggestive evidence for a significant reduction in the hepatocytes from the male SAC-KO mice compared to the male WT, which suggests reduced activity of the mTOR pathway. In addition, no change was found in either the amount of total protein or the number of phosphorylated components of the mTORC1 complex, principally of Raptor and Pras40 (Figure 4B–E). 

To better understand the effect of the hepatocyte-specific deletion of the Hh pathway on mTOR signalling and the possible involvement of pathways closely connected to mTOR, we performed an Akt signalling phosphorylation array of male SAC-WT and SAC-KO mice (Figure S3). This array revealed a trend for reduced phosphorylation (<0.8) of Akt, glycogen synthase kinase-3 alpha (GSK3a), and RAF proto-oncogene serine/threonine-protein kinase (Raf-1) in the male SAC-KO. In contrast, the phosphorylation of 4E-BP1 (p4E-BP1), glycogen synthase kinase-3 beta (GSK3b), cyclin-dependent kinase inhibitor 1B (p27), p70S6 and, especially, the ribosomal S6 kinases 1 and 2 (RSK1/2) was tendency increased (>1.2). No alteration (0.8–1.2) was found for the AMP-activated protein kinase catalytic subunit alpha (AMPKa), Bcl2-associated agonist of cell death (BAD), mitogen-activated protein kinase 3 (ERK), mTOR (0.86), cellular tumour antigen p53 (p53), 3-phosphoinositide-dependent protein kinase 1 (PDK1), Pras40, phosphatidylinositol 3,4,5-trisphosphate 3-phosphatase, dual-specificity protein phosphatase (PTEN) or 40S ribosomal protein S6 (RPS6).

As IPA predicted the inhibition of autophagy in the SAC-KO mice, we examined the amount of p62 in the hepatocytes. p62 binds ubiquitinylated proteins and recruits them to the autophagosomes, where they are degraded, therefore p62 accumulates when autophagy is impaired [27,28]. We performed Western blotting to analyse the amount of p62 in the hepatocytes from the SAC mice and detected suggestive evidence for a significant increase in the p62 levels of the SAC-KO hepatocytes from male mice whereas females show only a tendency for upregulation of p62 (Figure S4), which partially confirmed the prediction made by the IPA software.

Although the activity of the mTOR pathway is mainly regulated through the phosphorylation of its components, we also looked at the mRNA levels of the mTOR signalling molecules. Hepatocytes from the female SAC-KO mice showed significant decreased mRNA expression levels of the mTORC1 components *Mtor*, *Deptor* and *Pras40* and the downstream molecule *4e-bp1*, which suggested the repression of the mTORC1/4E-BP1 signalling cascade (Figure S5). In contrast, the mRNA level of the mTORC2 compartment *Rictor* showed suggestive evidence for a significant upregulation. However, the hepatocytes from the male SAC-KO mice showed no alterations in the mRNA levels of mTOR signalling molecules, except for a small decrease in *Mlst8*, which was not altered in the female mice. The mTORC1 compartment *Raptor* and the downstream kinase *P70s6* exhibited no changes in the mice of either sex. 

Altogether, these results support the hypothesis that hepatic Hh pathway influences mitochondrial metabolism and autophagy through the stimulation of mTOR signalling.

### 3.3. Cyclopamine and Rapamycin Have a Synergistic Effect on Mitochondrial Metabolism and mTOR Signalling Activity

**Figure 5:**
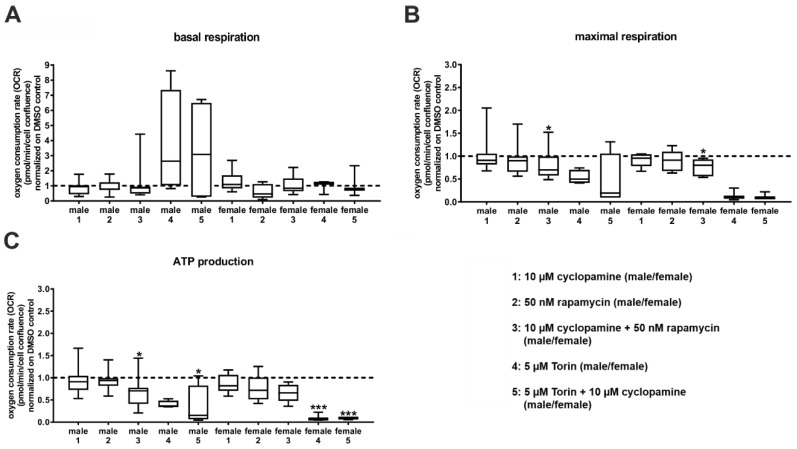
Combinatory effects of Hedgehog and mechanistic target of rapamycin inhibitors on mitochondrial respiration. Mito stress test analysis of primary hepatocytes from C57BL/6N mice. The oxygen consumption rate (OCR) was measured with the Seahorse analyser. The dashed line shows the DMSO control. (**A**): Basal respiration. (**B**): Maximal respiration. (**C**): Adenosine triphosphate (ATP) production. Significance was calculated with one-way ANOVA for repeated measurements. Stars show the significance to DMSO control. * *p* ≤ 0.05, *** *p* ≤ 0.001; *n* = 3–4.

To examine whether the Hh and the mTOR pathways interact with each other to stimulate mitochondria, we incubated primary hepatocytes from C57BL/6N mice for 24 h with the Hh inhibitor cyclopamine, the mTORC1 inhibitor rapamycin and the mTORC1/2 inhibitor Torin, as well as combinations of them. Subsequently, we analysed the hepatocytes using a Seahorse analyser. The basal respiration remained constant under all conditions (Figure 5A). However, male mouse hepatocytes showed a high variability when incubated with Torin and Torin combined with cyclopamine. Additionally, the incubation of the female mouse hepatocytes with rapamycin showed a minor decline. Interestingly, neither cyclopamine nor rapamycin had an effect on mitochondrial respiration in the male or female mice (Figure 5B,C). However, the combination of both inhibitors showed suggestive evidence for a significant reduction of the maximal respiration and the ATP production rates in both the male and female mouse hepatocytes. Additionally, the combination of cyclopamine and rapamycin slightly decreased the spare respiratory capacity and the non-mitochondrial oxygen consumption in both sexes, but these results were not significant (Figure S6). Torin had a significantly high impact on the non-mitochondrial oxygen consumption in the hepatocytes extracted from both sexes, with a greater impact on the ATP production in the female mouse hepatocytes (Figure 5; Figure S6). In addition, Torin also tendentially reduced the maximal respiration and the spare respiratory capacity in the hepatocytes from both sexes (Figure 5; Figure S6). However, the combination of cyclopamine and Torin had no additional effect in the hepatocytes from the mice of either sex.

**Figure 6:**
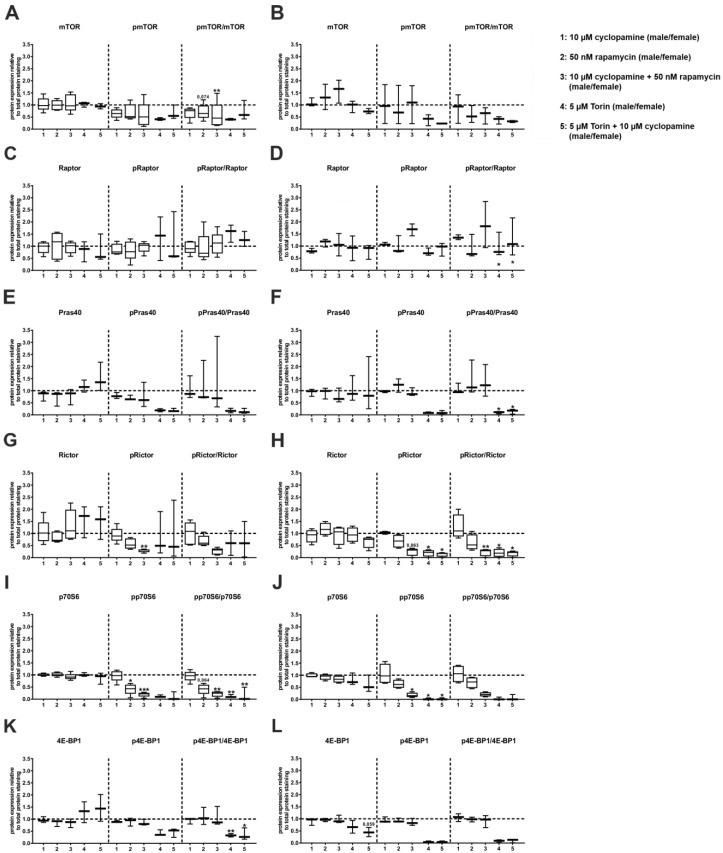
Protein expression and phosphorylation state of the mechanistic target of rapamycin (mTOR) signalling after inhibition of Hedgehog and/or mTOR signalling. Primary hepatocytes from C57BL/6N mice were isolated and incubated for 24 h with 10 µM cyclopamine, 50 nM rapamycin, 5 µM Torin and combinations of them. (**A**,**L**): Densitometric evaluation of the band intensities of the Western blots normalized on the total protein staining, relative to DMSO control (dashed line). (**A**,**B**): mTOR and phosphorylated (p) mTOR (Ser2448) of male (**A**) and female (**B**) hepatocytes. (**C**,**D**): Raptor and pRaptor (Ser792) of male (**C**) and female (**D**) hepatocytes, (**E**,**F**): Pras40 and pPras40 (Thr246) of male (**E**) and female (**F**) mice. (**G**,**H**): Rictor and pRictor (Thr1135) of male (**G**) and female (**H**) hepatocytes. (**I**,**J**): p70S6 and pp70S6 (Thr389) of male (**I**) and female (**J**) hepatocytes. (**K**,**L**): 4E-BP1 and p4E-BP1 (Thr37/46) from male (**K**) and female (**L**) hepatocytes. Significance was calculated with one-way ANOVA for repeated measurements. Stars show the significance to Dimethyl sulfoxide (DMSO) control. * *p* ≤ 0.05, ** *p* ≤ 0.01, *** *p* ≤ 0.001; *n* = 3–5.

To understand the interaction between both pathways, we looked at the phosphorylation state of several mTOR pathway molecules using Western blot analysis. Here, we attained results similar to those obtained in the Seahorse analysis. The total amount of mTOR, Raptor, Pras40, Rictor, p70S6 and 4E-BP1 did not change in any of the treatments (Figure 6, Figure S7). Cyclopamine alone had no effect on the phosphorylation of the mTOR signalling molecules. The addition of rapamycin reduced the phosphorylation of p70S6 (pp70S6) in the male C57BL/6N hepatocytes, as expected, and the phosphorylation of Rictor (pRictor) in the male mouse hepatocytes and pp70S6 in the female mouse hepatocytes showed a similar downward trend; however, the combination of both inhibitors significantly reduced the pp70S6 and showed suggestive evidence for significant reduction of pRictor in male mouse hepatocytes. In female mouse hepatocytes, we detected suggestive evidence for significance of reduced pp70S6. Additionally, the ratio of pmTOR to mTOR was significantly reduced in the male mouse hepatocytes. In contrast, the phosphorylation state of the mTORC1 compartments Raptor and Pras40 remained unchanged in the hepatocytes from both male and female mice upon the treatment with cyclopamine, rapamycin or both. The addition of Torin led to a suggestive evidence of a significant decrease in the phosphorylation of p70S6 in the female mouse hepatocytes. The ratio of the phosphorylated Pras40 protein to the total amount of protein showed suggestive evidence for significant reduction in female mouse hepatocytes. In the male mouse hepatocytes, the ratio of pp70S6 to total protein was significantly reduced and the ratio of p4E-BP1 to total protein showed suggestive evidence for a significant reduction with Torin. However, a reducing trend was also measured for the phosphorylation of mTOR, Pras40 and 4E-BP1 in the hepatocytes from both sexes. Only the phosphorylation state of Raptor remained unchanged upon the addition of Torin to the culture. The addition of cyclopamine and Torin led to similarly results. 

In addition, we examined the expression level of several genes associated with Hh signalling and some involved in mitochondrial metabolism (Figures S8 and S9). We did not detect any significant change in the mRNA expression levels of the genes involved in canonical Hh signalling, such as *Gli1*, *Ptch1*, *Smo* or *Fu*. Furthermore, the treatment with cyclopamine showed only a modest non-significant downregulation of *Gli1* and *Ptch1*. Only the expression of *Gsk3β* showed a significant increase after the addition of Torin in the culture of female C57BL/6N hepatocytes. The mRNA level of mitochondrial genes such as *Slc25a1*, *Slc25a15*, *Slc25a20* and *Slc25a45* did not change. Only the expression of *Slc25a4* showed suggestive evidence for a significant reduction with cyclopamine and rapamycin treatment and was significantly reduced during the treatment with Torin and Torin in combination with cyclopamine in the male mouse hepatocytes. However, the gene expression patterns of *Gsk3β*, *Slc25a1*, *Ccnd1* in the hepatocytes from both sexes and that of *Mtor* and *Sufu* in the female mouse hepatocytes showed the same synergistic inhibition of cyclopamine and rapamycin, corroborating the findings of the Western blot and Seahorse analyses. The combined treatment of cylcopamine and rapamycin led to suggestive evidence of a significant decrease in the expression of *Sufu* in the female mouse hepatocytes.

In summary, the simultaneous inhibition of Hh and mTOR signalling using cyclopamine and rapamycin treatments led to a synergistic repression of mTOR signalling and energy metabolism. However, the addition of cyclopamine to the hepatocytes treated with Torin had no further effect. 

## 4. Discussion

Our study shows that the hepatocyte-specific repression of canonical Hh signalling through the deletion of *Smo* has an impact on mTOR signalling, autophagy and mitochondrial respiration and therefore influences the energy metabolism of the hepatocytes. Furthermore, we demonstrate the interaction of both pathways in healthy hepatocytes via the synergistic effect of cyclopamine and rapamycin on the inhibition of the mTOR pathway. Additionally, we detected significant differences in the regulation of different aspects of energy metabolism in the hepatocytes from male and female mice, as discussed below.

### 4.1. Hh/mTOR Crosstalk in Hepatocytes

Currently the interaction of Hh and mTOR signalling is mainly examined in cancerous tissues. The dysregulation of both pathways plays a role in the development and progression of a variety of cancers, and therefore, Hh and mTOR are targets for clinical treatments. However, the number of tumour types in which Hh and mTOR interact is as large as the variability of their postulated mutually interactions. Some studies have shown that mTOR signalling can directly and indirectly regulate the activation of Gli factors, stimulating non-canonical Hh signalling [13,29]. Others have suggested that mTOR signalling is required for Hh signalling [30,31]. In contrast to the mechanisms described for different cancer types, our female mice with hepatocyte-specific Hh KO exhibit reduced mTOR activity, as was shown through reduced ATP production and mTOR-related gene expression. In addition, low levels of phosphorylated mTOR were found in the male SAC-KO mouse hepatocytes and accumulated p62 was detected in the hepatocytes from both sexes. The accumulation of p62 is a sign for impaired autophagy and additionally is related to chronic liver diseases [32]. Furthermore, it is well known that the mTOR pathway regulates autophagy [2]; therefore, these findings indicate that the absence of Hh signalling can regulate mTOR activity. In contrast, in their 2008 study, Parathath et al. described Hh activation via SHH-induced inhibition of mTOR/p70S6 signalling [33]. However, in the current study, the Akt signalling phosphorylation array analysis revealed increased phosphorylation of the two mTORC1 downstream molecules p70S6 and 4E-BP1 in the male SAC-KO mouse hepatocytes. According to this finding, the IPA software predicted the activation of the mitochondrial transport chain and the mTOR pathway in the male SAC-KO mice, which confirmed the results from Parathath et al. 2008 [33], although the ratio of pmTOR-to-mTOR was reduced. In contrast, the phosphorylation of Akt was reduced and the phosphorylation of RSK1 and RSK2 was elevated. Both findings indicate the inhibition of mTOR signalling [34]. 

**Figure 7:**
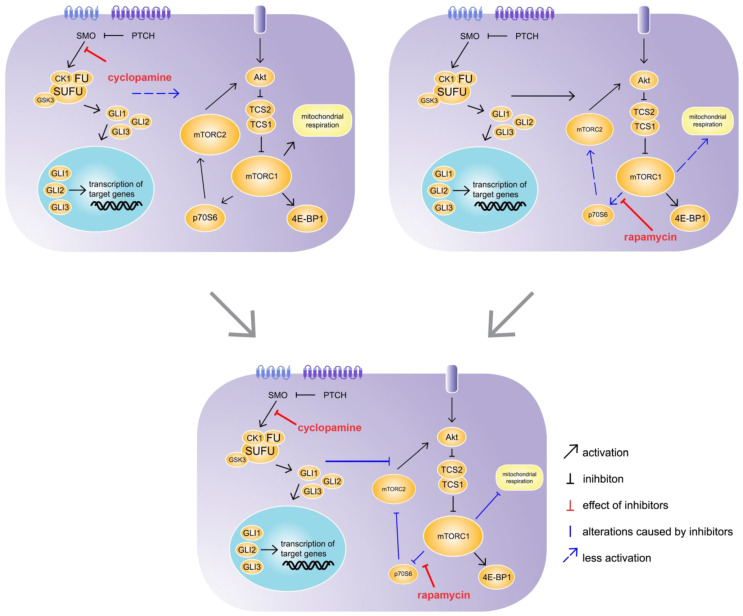
Synergistic effect of cyclopamine and rapamycin. C57BL/6N hepatocytes were treated with cyclopamine and/or rapamycin for 24 h. The treatment with cyclopamine had no effect on mechanistic target of rapamycin (mTOR) signalling, whereas the treatment with rapamycin had weak inhibitory effects on p70S6 downstream signalling and mitochondrial respiration. However, the simultaneous treatment with cyclopamine and rapamycin exhibited strong inhibitory effects on p70S6 downstream signalling as well as mitochondrial respiration, revealing an impact of Hh signalling on the mTOR pathway. SMO: Smoothened, PTCH: Patched, CK1: Casein kinase 1, FU: Fused, SUFU: Suppressor of Fused, GSK3: Glycogen synthase kinase 3, GLI1-3: glioma-associated oncogene family zinc finger 1-3, Akt: Protein kinase B, TCS: Tuberous sclerosis complex, mTORC1/2; mechanistic target of rapamycin complex 1/2, 4E-BP1: Eukaryotic translation initiation factor 4E-binding protein 1, p70S6: Ribosomal protein S6 kinase beta-1.

While cyclopamine binds to the receptor SMO and thus inhibits the Hh pathway in hepatocytes at the same position where it was blocked in the SAC-KO mice, it did not have the same effect in the short time of 24 h in vitro on hepatocytes from C57BL/6N mice. In the SAC mice, the Hh pathway is blocked in the hepatocytes 9 days after fertilization [18]. Therefore, the hepatocytes lacked Hh signalling for 3 months before they were analysed. During this time these hepatocytes were embedded in their natural environment prior to analyses. In contrast, the Hh signalling in the hepatocytes from the C57BL/6N mice was blocked for only 24 h after preparation in the 2D cell culture; therefore, they were exposed to impaired Hh signalling for a much shorter time and were not exposed to systemic effects. Unfortunately, we could not detect significant downregulation of *Gli1* and *Ptch1* in primary hepatocytes treated with cyclopamine for 24 h as it is known for numerous other cell types [35,36]. Several reasons for this apparent discrepancy are discussed below. Regarding mTOR signalling, in combination with rapamycin, cyclopamine had an even stronger effect on mTOR signalling compared to rapamycin alone. This synergistic effect was also observed in the 2015 study by Zuo et al. using a xenograft mouse model for biliary tract cancer treated with a combination of vismodegib, which is also a SMO inhibitor, and rapamycin [37]. However, our results revealed an interaction of Hh and mTOR signalling in healthy liver hepatocytes. Interestingly, the effect of the treatment of the primary hepatocytes with Torin exceeded the impact of cyclopamine in combination with rapamycin on mTOR signalling in most cases; however, the addition of cyclopamine to Torin had no added effect. In contrast to rapamycin, Torin inhibits the complete downstream signalling pathways of mTORC1 and mTORC2. Because the Torin-induced inhibition was not enhanced by adding cyclopamine, Torin may block the point where these pathways interact. mTORC2 is only indirectly affected by rapamycin via a feedback mechanism involving p70S6 and Akt. We suggest that Hh has an activating effect on mTORC2, which attenuates the inhibitory effect of rapamycin on mTOR signalling through the Akt feedback mechanism (Figure 7). This idea is supported by the results from a 2008 by Ikenoue et al., which showed that the mTORC2-mediated phosphorylation of Akt was required for the maximum level of Akt-activated signalling to be induced [38]. Therefore, cyclopamine seems to prevent Hh from activating mTORC2, revealing the full strength of the inhibition through rapamycin. However, so far, we cannot definitively exclude possible off-target effects of cyclopamine or the involvement of the non-canonical Hh pathway. Therefore, further studies are needed to clarify these issues.

### 4.2. Hh/mTOR Crosstalk Influences Mitochondrial Metabolism and Therefore the Energy State of the Cell

It is well known that the mTOR pathway regulates mitochondrial metabolism. The downstream signalling of mTORC1 influences mitochondrial biogenesis, morphology and ATP production capacity [3,39,40]. In contrast, the indications to date suggest that the Hh pathway is not closely connected with mitochondrial metabolism. Interestingly, hepatocytes from the female SAC-KO mice showed suggestive evidence for significantly reduced ATP production, which was consistent with the prediction of the IPA software based on proteome data, thereby linking repressed Hh signalling with mitochondrial function. Our results were partly consistent with a 2017 study from Yao et al. showing that Hh activation based on SHH led to an increase in mitochondrial mass, elongation, membrane potential and respiratory activity in hippocampal neurons [41]. However, we did not detect any changes in the mitochondrial mass of the hepatocytes from the SAC-KO mice. Our results were consistent with those from the study by Kalim et al., who showed that mTOR-related changes in mitochondrial mass were dependent on Raptor [42], which was unchanged in our SAC hepatocytes. In addition, the hepatocytes from the SAC-KO mice exhibited increased mitochondrial membrane potential, which also is an opposite finding than that of the Yao et al. study in 2017 [41]. However, it is known that inhibition of ATP synthase can lead to increased mitochondrial membrane potential [43]. Therefore, our results suggest that repressed Hh signalling leads to the inhibition of ATP synthase, a supposition that should be examined in further studies. Moreover, we detected elevated mRNA levels of mitochondrial carrier proteins involved in the citric acid cycle in the hepatocytes from the male but not from the female SAC-KO mice, although male mouse hepatocytes showed no alteration in ATP production. In the female SAC-KO mouse hepatocytes, we detected an increase in the gene expression of the adenine nucleotide translocator *Slc25a4*. This upregulation could have been a response to the reduced ATP levels and correlates with the striking increase in the proton leak [44]. Additionally, it is known that the proton leak depends on the mitochondrial membrane potential [43]. Therefore, the hyperpolarization of the mitochondria may have also contributed to the increased proton leak. However, the question remains: how does Hh signalling regulate mitochondrial metabolism? One possible explanation may involve the direct interaction of Hh with mTOR signalling. This idea is supported by the synergistic effect of rapamycin and cyclopamine on the inhibition of the maximal respiration and the ATP production rates of the hepatocytes. As mTOR is the main regulator for mitochondrial metabolism, the synergistic inhibition of mTOR signalling through cyclopamine and rapamycin affects the oxidative phosphorylation in mitochondria. Nevertheless, it is possible that cyclopamine influences oxidative phosphorylation independently from the canonical Hh signalling pathway, as it was postulated by Kalainayakan et al. in 2019 [45]. This assumption is supported by the fact that no significant changes were observed in the gene expression of *Gli1* and *Ptch1* after exposure to cyclopamine. However, the reasons for this observation may be manifold. Firstly, as reported by Ozretić et al. 2017 in tumour cells, the strength of the inhibitory effect of cyclopamine on the mRNA levels of *Gli1* and *Ptch1* may depend strongly on the cell type [46]. Moreover, specifically in hepatocytes, we recently found that *Gli1* is part of a Gli-network and behaves different than in other cell types [25]. Furthermore, it is known that the expression of both genes is subject to pronounced diurnal oscillation [11]. Thus, measurements at a single time point may not be the best choice for estimating Hh pathway activity. In addition, it would be interesting to investigate possible off-target effects of cyclopamine. Therefore, further studies are needed to reveal the exact molecular mechanism of the impact of Hh signalling on mitochondrial metabolism.

### 4.3. The Sensitivity of Energy Metabolism to Hepatocyte-Specific Hh Signalling is Sex-Specific

The liver is one of the most dimorphic organs between sexes. The differences play an important role in medical treatment as many drugs are metabolized in the liver in a sex-specific manner [47]. Although this phenomenon has been known since the 1960s, most of the molecular mechanisms have yet to be revealed. Therefore, we conducted most of the experiments in this study in both male and female mice to examine possible sex-specific differences in the Hh and mTOR signalling pathways. As was previously observed, the Hh pathway influences the development of sexual organs by regulating androgen metabolism [48,49,50]. Therefore, it appears obvious that Hh also regulates other pathways in a sex-specific manner. Our results showed that the hepatocyte-specific KO of Hh had a stronger impact on the energy metabolism in the female mouse compared to male mouse hepatocytes. The female mouse hepatocytes showed suggestive evidence for significantly decreased ATP production in the Seahorse analysis, as was predicted by the IPA software based on the female mouse proteome. This ATP reduction was the result of a higher proton leak and higher mRNA levels of *Slc25a4* expression. None of these observations were made in the hepatocytes from the male SAC-KO mice. However, the mitochondrial membrane potential was elevated in the hepatocytes from both sexes. In contrast to the sexual dimorphism in vivo, we did not observe differences in vitro, although rapamycin is known to exhibit sex-specific effects in cardiac and renal tissue [51,52,53]. The only sex-specific differences we detected were based on rapamycin effects on transcriptional level. The mRNA expression of *Sufu* in the hepatocytes from C57BL/6N female mice showed a similar synergistic inhibition upon cyclopamine and rapamycin treatment as was detected at the protein level when p70S6 was in the phosphorylated state in both sexes. Interestingly, in the male mouse hepatocytes, the mRNA levels of *Slc25a4* were reduced upon all inhibitor treatments. These results suggest that Hh signalling has sex-specific effects only in a systemic context, which is likely caused by its effect on androgen metabolism and steroidogenesis [49].

## 5. Conclusions

The Hh pathway can influence autophagy and energy metabolism, especially oxidative phosphorylation, by promoting mTORC2 signalling in hepatic tissue. Female mice seem to be more sensitive towards the effect of Hh signalling in terms of energy metabolism. These findings should be considered, as therapeutic treatments targeting the Hh pathway seem to be a promising therapy for different cancer types [54,55]. The sex-specific effect of Hh inhibitors on energy metabolism could make a difference in the survival of male and female patients, which is often reduced by aggressive therapies. Our results could, therefore, be used as a basis for further studies focusing on sex-based therapies, as it is well known that men and women have different needs concerning medical treatments [56]. Furthermore, cyclopamine and rapamycin established a synergistic inhibition of mTOR signalling. These results reveal new insights into the regulating network of hepatic energy metabolism. The synergistic effect of cyclopamine and rapamycin should be considered in the search for more effective therapeutic interventions for cancerous diseases like the hepatocellular carcinoma (HCC). Nevertheless, further studies are necessary to expand the knowledge on the molecular interactions of these important morphogenic pathways.

## Figures and Tables

**Table 1:** Primer sequences for qRT-PCR. f: forward primer; r: reversed primer; the primer for Gli1 was purchased from Qiagen (Mm_Gli1_1_SG QuantiTect Primer Assay).

Gene Names	Primer Sequences	
***Actb***	F	CATCCGTAAAGACCTCTATGCCAAC
	R	ATGGAGCCACCGATCCACA
***Ccnd1***	F	GAGACCATTCCCTTGACTGC
	R	TGGTCTGCTTGTTCTCATCC
***Deptor***	F	TCTCAGGAGACGCATGACAG
	R	AGATTTGGGGTTGCAGAGC
***4E-bp1***	F	GGGACTACAGCACCACTCC
	R	CTCATCGCTGGTAGGGCTAG
***Fu***	F	TGCCTCTCAGCCTTCTTAGG
	R	TAAGAGCGCCCCATACCA
***Gsk3-β***	F	CCGTCTGCTGGAGTACACAC
	R	GAGCATGTGGAGGGATAAGG
***Mlst8***	F	CAGAGTGCAGAGCGTGTGTT
	R	GCCTGCAGTTGCTAAGATGA
***Mtor***	F	AATGAGGGCCGGAGACAT
	R	TGTTGTCAAAGAAGGGCTGA
***Pras40***	F	GAGGAAGATGAGGACGAGCC
	R	TGGGTAGGCAGGGCTGTG
***Ptch1***	F	ACTCCAAAAGAAGAAGGCGC
	R	CCAGAAGCAGTCCAAAGGTG
***Raptor***	F	GGCCTGCCTCCAGGGAAACC
	R	GATCCTTCCCAGGCAAATGG
***Rictor***	F	TTCCACTACAGACACAGTCCAGAT
	R	TGGCTAGAAATCGTGCTTCTC
***S6k1***	F	GGGGAGTTGGACCATATGAAC
	R	TCTTCCCAGTATTTGCTCCTG
***Slc25a1***	F	GGCACACAAATACCGGAAC
	R	AATACGATGGCCACATCCAG
***Slc25a4***	F	TCGTAGGATGATGATGCAGTCT
	R	TTGGCTCCTTCATCTTTTGC
***Slc25a15***	F	GGCACTGTTTTTGGCCTATG
	R	ACCATGCTTCCAATTGGTTC
***Slc25a20***	F	AAATCTCCAGAGGATGAACTTAGC
	R	CCTGTGGTGAACACACCAGATA
***Slc25a47***	F	GGACTCTACAAGGGCAGCTC
	R	AAAGTAGGTGGCAAAGGAGTGA
***Smo***	F	GCAAGCTCGTGCTCTGGT
	R	GGGCATGTAGACAGCACACA
***Sufu***	F	CTTCCAGTCAGAGAACACCT
	R	TTGGGCTGAATGTAACTC
***Ywhaz***	F	TTACTTGGCCGAGGTTGCT
	R	TGCTGTGACTGGTCCACAAT

## Supplementary Materials

The following are available online at https://www.mdpi.com/2073-4409/9/8/1817/s1, Figure S1: Analysis of the cell energy phenotype and mRNA expression of mitochondrial genes from primary hepatocytes of SAC mice., Figure S2: Representative example of flow cytometric analysis of mitochondrial mass and membrane potential., S3: Akt signalling phosphorylation array of hepatocytes from male SAC mice., Figure S4: Impact of hepatocyte-specific SAC-KO on autophagy., Figure S5: RNA expression of mTOR-related genes in SAC mice., Figure S6: Combinatory effects of Hh and mTOR inhibitors on mitochondrial respiration., Figure S7: Representative examples of the Western blots of male and female C57BL/6N mice after treatment with cyclopamine, rapamycin and Torin., Figure S8 and S9: qPCR analysis of male and female hepatocytes treated with cyclopamine, rapamycin and Torin for 24 h., Table S1: IPA analysis of the oxidative phosphorylation of male SAC mice., Table S2: IPA analysis of the oxidative phosphorylation of female SAC mice., Table S3: IPA analysis of the mTOR pathway in male SAC mice., Table S4: IPA analysis of the mTOR pathway in female SAC mice. Western blots, original pictures of all Western blots of the SAC and C57BL/6N mice with their densitometrical evaluation. The following are available online at https://seek.lisym.org/data_files/456?code=EU5FW714RhmQlWDxI%2Bpbt8D%2BRwPxsfbjbBjP5zdQ, proteome data of female SAC mice. The following are available online at https://seek.lisym.org/data_files/457?code=Vm2d5I0I4ni0SdaoeZln0vMDCrlNXofNB4Am%2FGV3, proteome data of male SAC mice.
