# Supplementary material for: Cyclopamine and Rapamycin Synergistically Inhibit mTOR Signalling in Mouse Hepatocytes, Revealing an Interaction of Hedgehog and mTor Signalling in the Liver
Source: Cells. 2020 Jul 31;9(8):1817. doi: 10.3390/cells9081817 (PMC7464279; doi:10.3390/cells9081817)
Supplement: Supplementary file 1 [file cells-09-01817-s001.pdf]

Supplementary Materials

Supplementary Figures

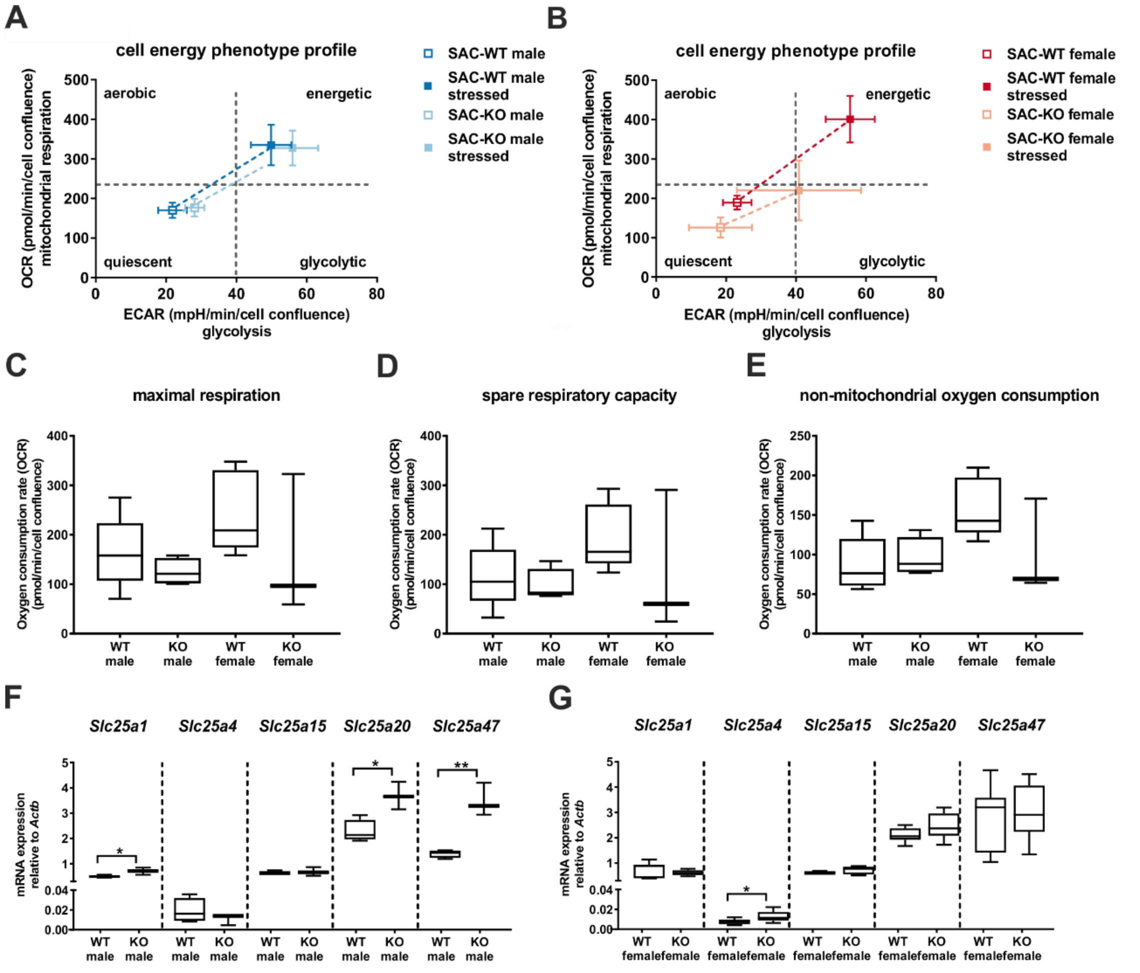

Figure S1: Analysis of the cell energy phenotype and mRNA expression of mitochondrial genes from primary hepatocytes of SAC mice. A-B: To determine the cell energy profile of hepatocytes from male (A) and female (B) SAC mice, hepatocytes were incubated with oligomycin and FCCP (stressed). C-E: Seahorse analysis Mito Stress Test of SAC mice. F-G: qPCR analysis of important genes of mitochondrial metabolism from male (F) and female (G) SAC mice. Error bars in A-B show SEM. Significance was calculated with multiple t-test. Stars show the significance to WT animals. * - P ≤ 0.05, ** - P ≤ 0.01, *** - P ≤ 0.001; N = 3-5.

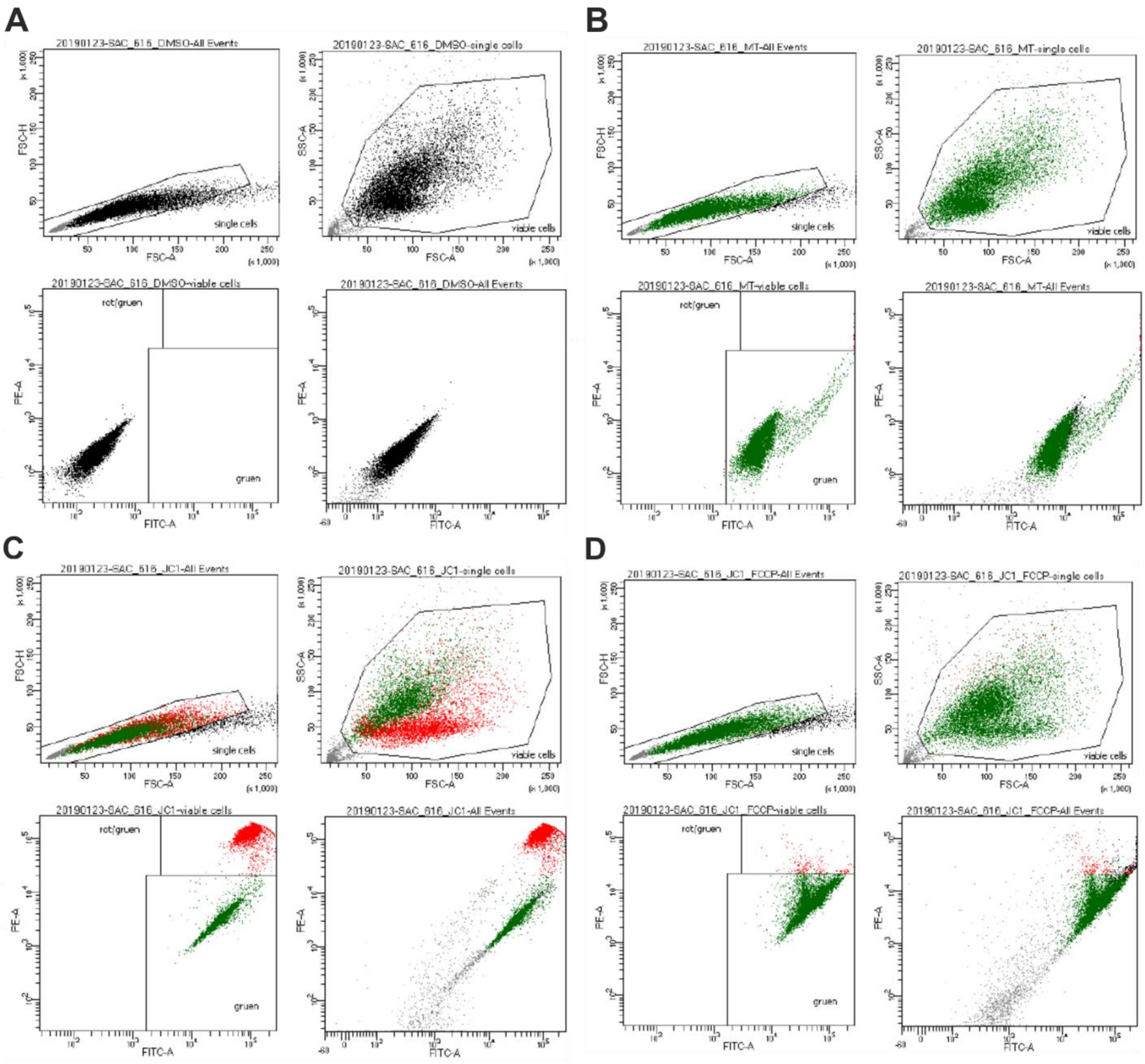

Figure S2: Representative example of flow cytometric analysis of mitochondrial mass and membrane potential. The figure shows representative data from one mouse. A: unstained negative control. B: Mito Tracker green staining for determination of mitochondrial mass. C: JC-10 staining for determination of mitochondrial membrane potential. D: negative control of JC-10 staining (addition of FCCP).

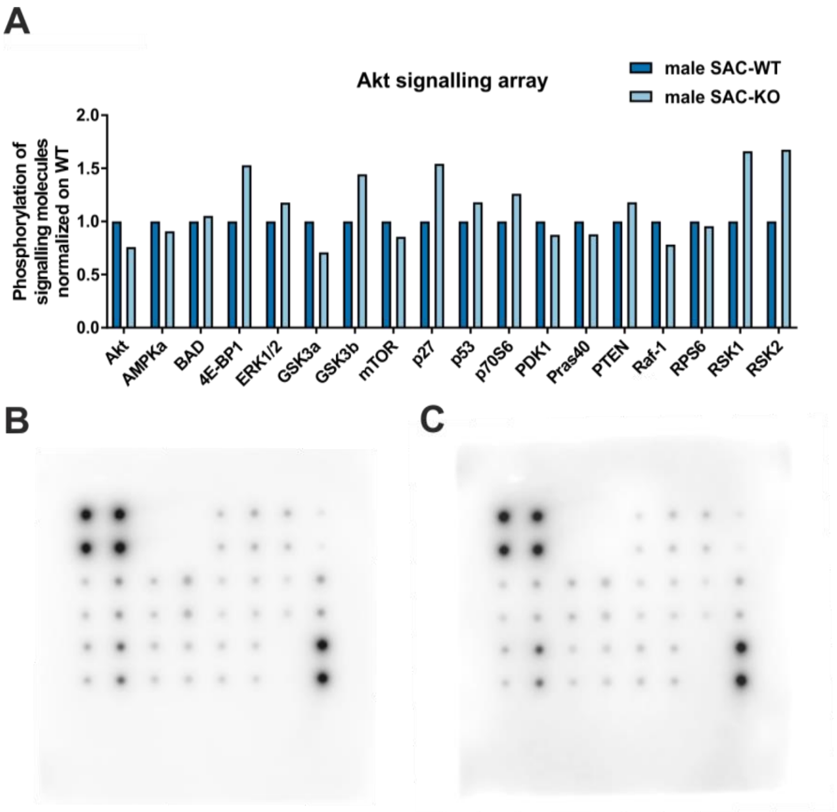

	1	2	3	4	5	6	7	8
1<br>2	POS	POS	NEG	NEG	Akt (p-Ser473)	AMPKα (p-Thr172)	BAD (p-Ser112)	4E-BP1 (p-Thr36)
3<br>4	ERK-1 (p-T202/Y204) ERK-2 (p-Y185-Y187)	GSK3α (p-Ser21)	GSK3β (p-Ser9)	mTOR (p-Ser2448)	p27 (p-Thr198)	p53 (p-Ser15)	p70S6 (p-Thr421/Ser 424)	PDK1 (p-Ser241)
5<br>6	Pras40 (p-Thr246)	PTEN (p-Ser380)	Raf-1 (p-Ser301)	RPS6 (p-Ser235/236)	RSK1 (p-Ser380)	RSK2 (p-Ser386)	NEG	POS

Figure S3: Akt signalling phosphorylation array of hepatocytes from male SAC mice. Primary hepatocytes from a male SAC-WT and -KO mouse were used for an Akt signalling phosphorylation array. A: Densitometric analysis of the spots according to manufacturer's instructions. B: Blot of male SAC-WT mouse. C: Blot of male SAC-KO mouse. POS – positive control, NEG – negative control, Akt - protein kinase B, AMPKα - 5'-AMP-activated protein kinase catalytic subunit alpha, BAD - Bcl2-associated agonist of cell death, 4E-BP1 - Eukaryotic translation initiation factor 4E-binding protein 1, ERK 1/2 - Mitogen-activated protein kinase, GSK3α - Glycogen synthase kinase-3 alpha, GSK3β - Glycogen synthase kinase-3 beta, mTOR – mechanistic target of rapamycin, p27 - Cyclin-dependent kinase inhibitor 1B, p53 - Cellular tumor antigen p53, p70S6 - Ribosomal protein S6 kinase beta-1, PDK1 - 3-phosphoinositide-dependent protein kinase 1, Pras40 - Proline-rich AKT1 substrate 1, PTEN - Phosphatidylinositol 3,4,5-trisphosphate 3-phosphatase and dual-specificity protein phosphatase, Raf-1 - RAF proto-oncogene serine/threonine-protein kinase, RPS6 - 40S ribosomal protein S6, RSK1/2 - Ribosomal protein S6 kinase alpha-1.

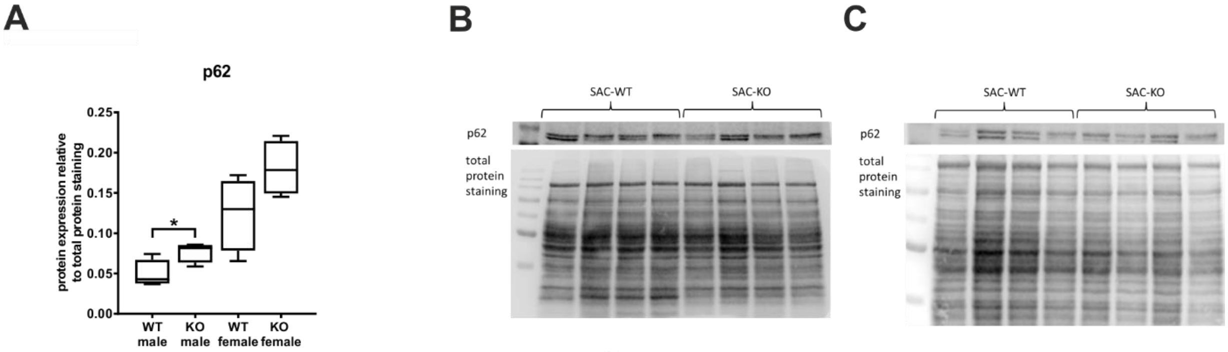

Figure S4: Impact of hepatocyte-specific SAC-KO on autophagy. A: Densitometric analysis of the Western blot of p62 in hepatocytes from male and female SAC mice. B: Western blot of p62 in male SAC mice. C: Western blot of p62 in female SAC mice. Significance was calculated with multiple t-test. Stars show the significance to WT animals. * - P ≤ 0.05, N = 4.

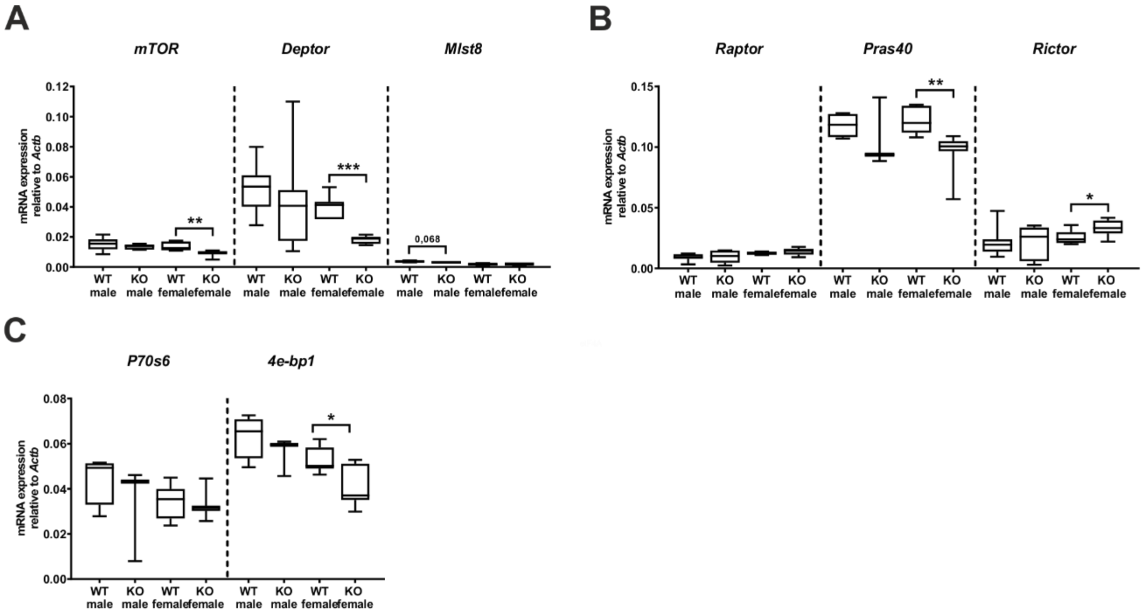

Figure S5: RNA expression of mTOR-related genes in SAC mice. A: mTORC1 and -C2 components *Mtor*, *Deptor* and *Mlst8*. B: mTORC1 components *Raptor* and *Pras40* and mTORC2 component *Rictor*. C: mTOR downstream signalling kinases *P70s6* and *4e-bp1*. Significance was calculated with unpaired t-test. Stars show the significance to WT animals. * - P ≤ 0.05, ** - P ≤ 0.01, *** - P ≤ 0.001; N = 3-8.

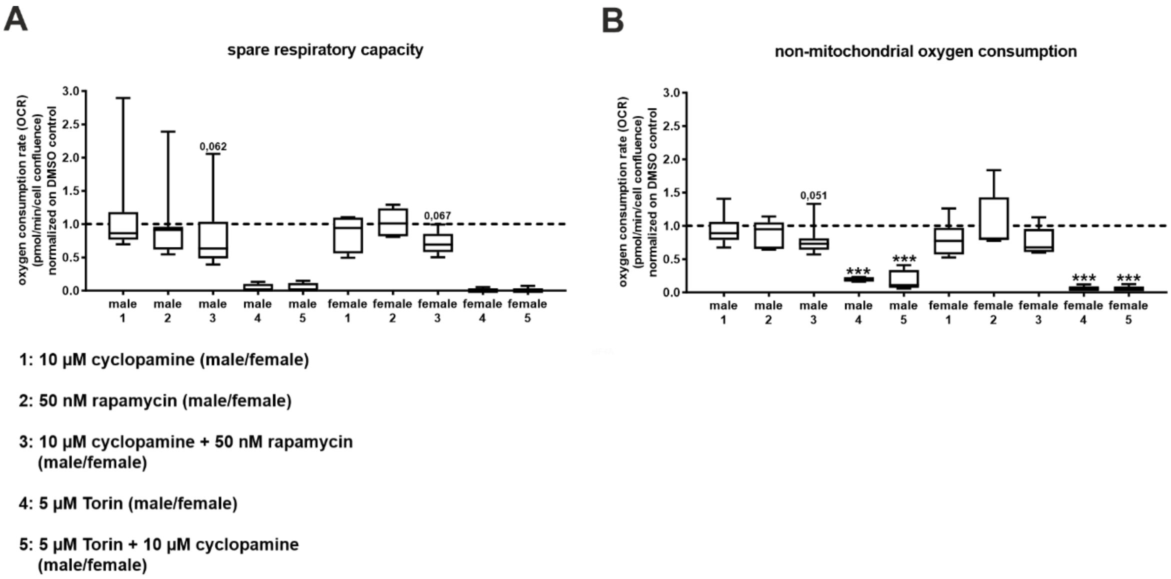

Figure S6: Combinatory effects of Hh and mTOR inhibitors on mitochondrial respiration. Mito stress test and cell energy phenotype analysis of primary hepatocytes from C57BL/6N mice. The oxygen consumption rate (OCR) was measured with the Seahorse analyser. The dashed lines show the DMSO control. A: Spare respiratory capacity. B: Non-mitochondrial oxygen consumption. Significance was calculated with one-way ANOVA for repeated measurements. Stars show the significance to DMSO control. * - P ≤ 0.05, ** - P ≤ 0.01, *** - P ≤ 0.001; N = 3-4.

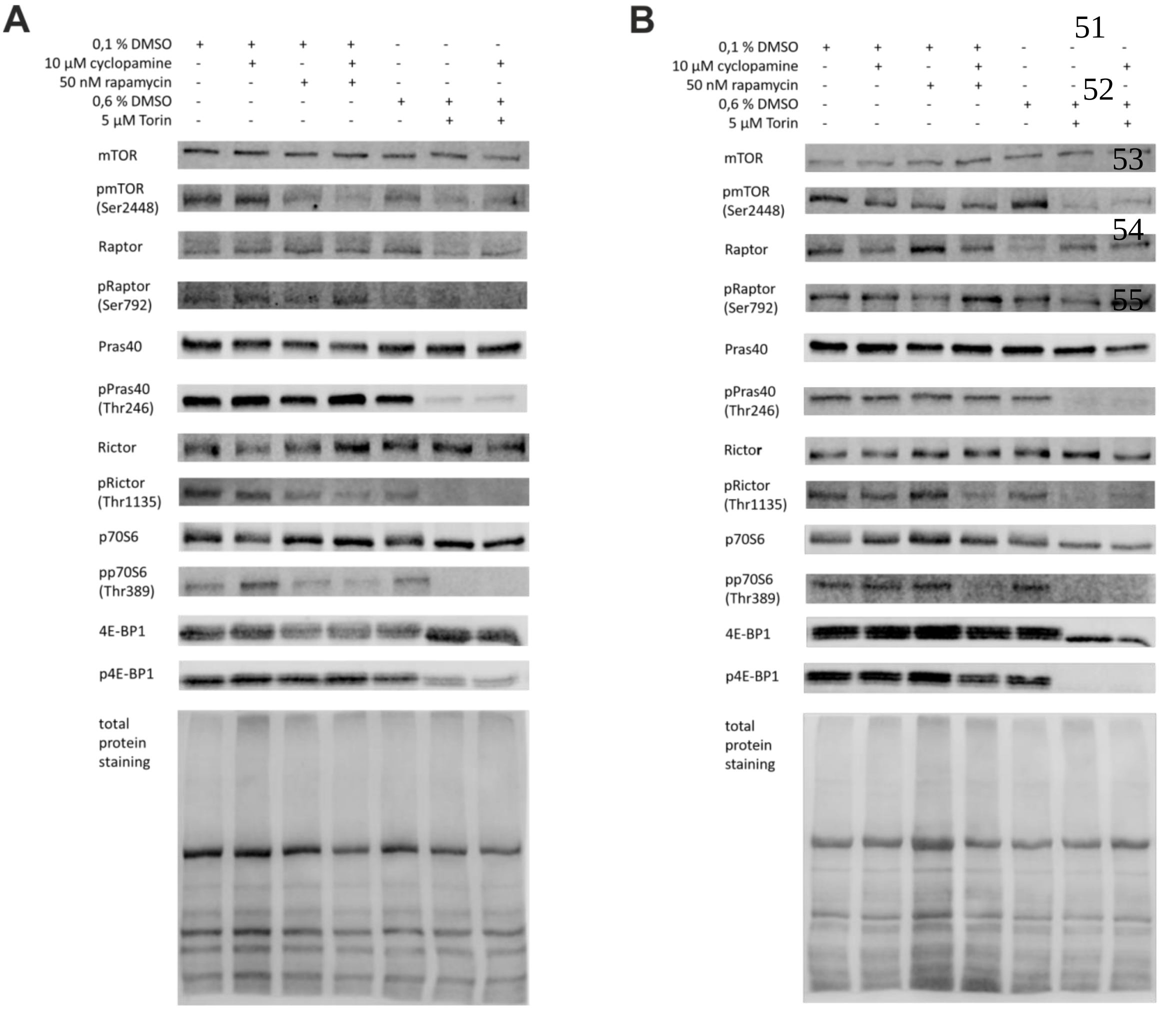

Figure S7: Representative examples of the Western blots of male and female C57BL/6N mice after treatment with cyclopamine, rapamycin and Torin. A: primary hepatocytes from male mice. B: primary hepatocytes from female mice.

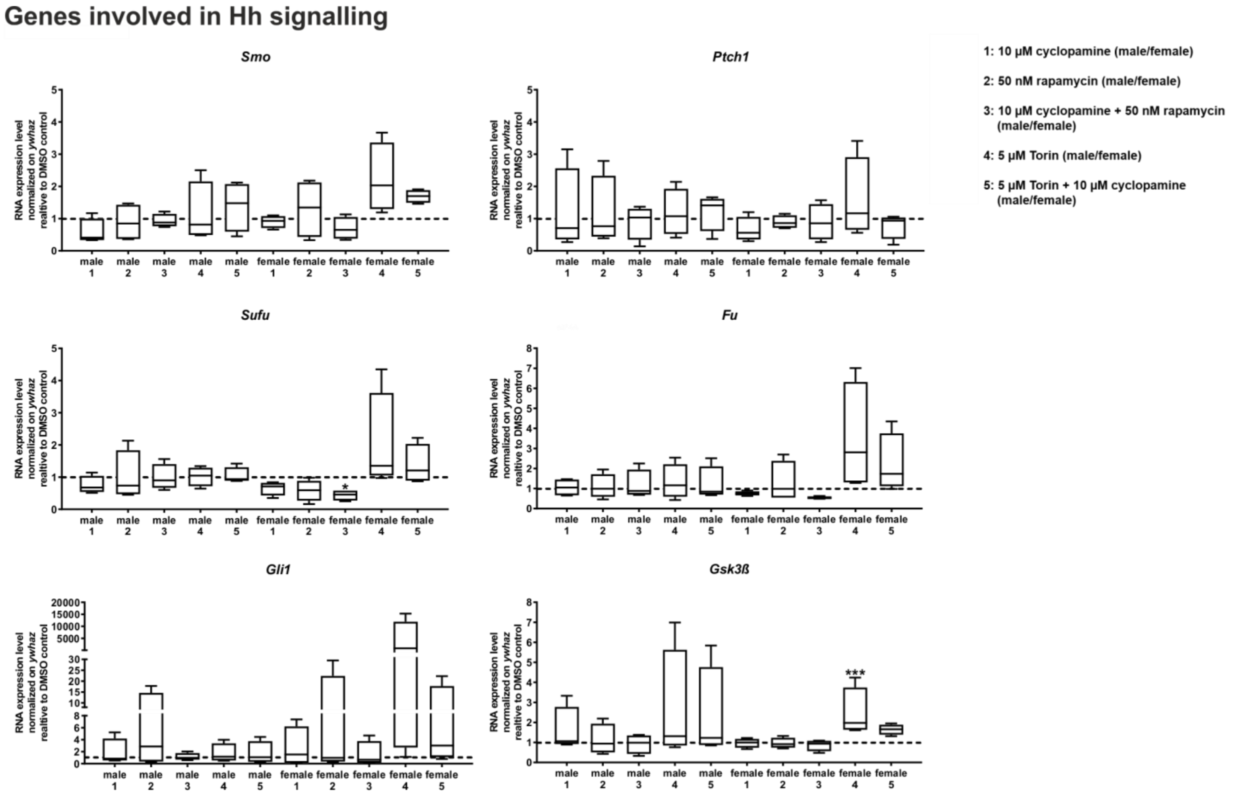

Figure S8: qPCR analysis of male and female hepatocytes treated with cyclopamine, rapamycin and Torin for 24 h. Results are shown relative to DMSO control (dashed line). Significance was calculated with one-way ANOVA for repeated measurements. Stars show the significance to DMSO control. * - P ≤ 0.05, ** - P ≤ 0.01, *** - P ≤ 0.001; N = 4

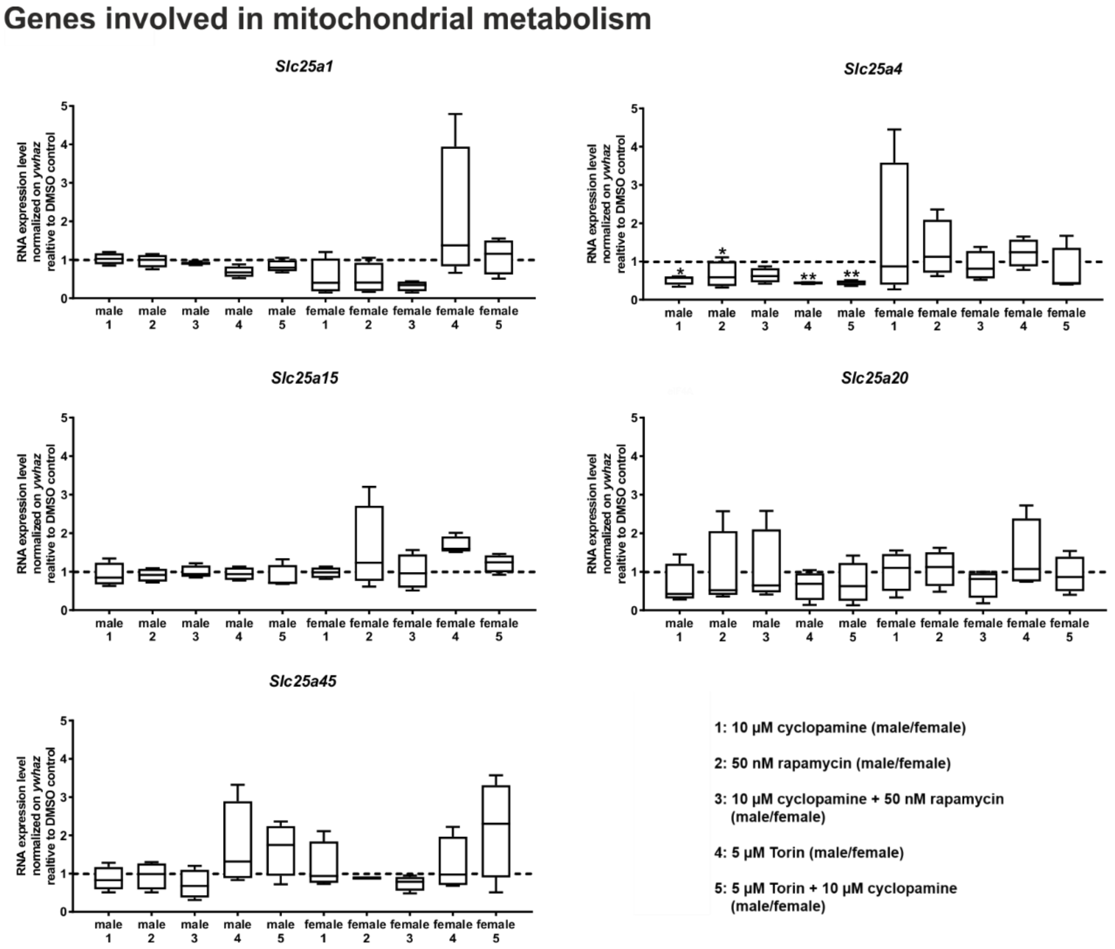

Figure S9: qPCR analysis of male and female hepatocytes treated with cyclopamine, rapamycin and Torin for 24 h. Results are shown relative to DMSO control (dashed line). Significance was calculated with one-way ANOVA for repeated measurements. Stars show the significance to DMSO control. * - $P \leq 0.05$, ** - $P \leq 0.01$, *** - $P \leq 0.001$; $N = 4$

Supplementary Tables

Table S1: IPA analysis of the oxidative phosphorylation of male SAC mice. Red color shows significantly upregulated proteins compared to WT mice.

Symbol	Entrez Gene Name	UniProt	Expression Fold Change	Expression p-value
**ATP5A1**	ATP synthase F1 subunit alpha	Q03265	1,058682801	0,212241
**ATP5B**	ATP synthase F1 subunit beta	P56480	1,022766332	0,640625
**ATP5C1**	ATP synthase F1 subunit gamma	Q8C2Q8	1,17285	0,0463706
**ATP5D**	ATP synthase F1 subunit delta	Q4FK74	-1,115838139	0,220541
**ATP5F1**	ATP synthase peripheral stalk-membrane subunit b	Q5I0W0	1,201342282	0,00826018
**ATP5J**	ATP synthase peripheral stalk subunit F6	P97450	-1,304911722	0,0944521
**ATP5J2**	ATP synthase membrane subunit f	P56135	1,042747041	0,83614
**ATP5L**	ATP synthase membrane subunit g	Q9CPQ8	1,373346649	0,0878789
**ATP5O**	ATP synthase peripheral stalk subunit OSCP	Q9DB20	1,197819315	0,0218166
**COX4I1**	cytochrome c oxidase subunit 4I1	A2RSV8	1,253557645	0,00489636
**COX5A**	cytochrome c oxidase subunit 5A	P12787	1,076923077	0,106636
**COX6B1**	cytochrome c oxidase subunit 6B1	P56391	1,160487117	0,16575
**COX7A2**	cytochrome c oxidase subunit 7A2	P48771	1,102265051	0,555348
**NDUFA10**	NADH:ubiquinone oxidoreductase subunit A10	Q99LC3	1,01743246	0,852348
**NDUFA11**	NADH:ubiquinone oxidoreductase subunit A11	G5E814	1,410237061	0,0420379
**NDUFA12**	NADH:ubiquinone oxidoreductase subunit A12	A0A0R4J275	1,487447699	0,00188871
**NDUFA13**	NADH:ubiquinone oxidoreductase subunit A13	Q9ERS2	1,283015152	0,000532561
**NDUFA2**	NADH:ubiquinone oxidoreductase subunit A2	Q9CQ75	1,571160291	0,0284502
**NDUFA4**	NDUFA4, mitochondrial complex associated	Q62425	1,741386456	0,021344
**NDUFA5**	NADH:ubiquinone oxidoreductase subunit A5	Q9CPP6	1,708982214	0,000554673
**NDUFA6**	NADH:ubiquinone oxidoreductase subunit A6	Q9CQZ5	1,476046516	0,052486
**NDUFA7**	NADH:ubiquinone oxidoreductase subunit A7	Q9Z1P6	1,613658743	0,000524843
**NDUFA8**	NADH:ubiquinone oxidoreductase subunit A8	Q9DCJ5	1,115959238	0,195955
**NDUFA9**	NADH:ubiquinone oxidoreductase subunit A9	A0A0R3P9C8	1,409242912	0,00416985
**NDUFAB1**	NADH:ubiquinone oxidoreductase subunit AB1	F6ZFT1	1,629851867	0,0175605
**NDUFB10**	NADH:ubiquinone oxidoreductase subunit B10	Q9DCS9	1,367406525	0,069593
**NDUFB3**	NADH:ubiquinone oxidoreductase subunit B3	Q9CQZ6	1,110904366	0,358154
**NDUFB4**	NADH:ubiquinone oxidoreductase subunit B4	Q9CQC7	1,01435099	0,890547
**NDUFB5**	NADH:ubiquinone oxidoreductase subunit B5	Q9CQH3	1,301350534	0,117685
**NDUFB7**	NADH:ubiquinone oxidoreductase subunit B7	Q9CR61	-1,138921732	0,522834
**NDUFB8**	NADH:ubiquinone oxidoreductase subunit B8	Q9D6J5	1,206208426	0,108491
**NDUFB9**	NADH:ubiquinone oxidoreductase subunit B9	Q9CQJ8	1,279164179	0,0499908
**NDUFS1**	NADH:ubiquinone oxidoreductase core subunit S1	Q91VD9	1,435179782	0,00107408
**NDUFS2**	NADH:ubiquinone oxidoreductase core subunit S2	Q91WD5	1,2595194	0,00122861

**NDUFS3**	NADH:ubiquinone oxidoreductase core subunit S3	Q9DCT2	1,031561462	0,761362
**NDUFS4**	NADH:ubiquinone oxidoreductase subunit S4	E9QPX3	1,427370367	0,0131995
**NDUFS6**	NADH:ubiquinone oxidoreductase subunit S6	P52503	1,409856632	0,0609841
**NDUFS7**	NADH:ubiquinone oxidoreductase core subunit S7	Q9DC70	1,322733173	0,0140408
**NDUFS8**	NADH:ubiquinone oxidoreductase core subunit S8	Q5M9P5	1,379349762	0,0302519
**NDUFV1**	NADH:ubiquinone oxidoreductase core subunit V1	D3YUM1	1,366963099	0,00507041
**NDUFV2**	NADH:ubiquinone oxidoreductase core subunit V2	Q9D6J6	1,220702055	0,0373881
**NDUFV3**	NADH:ubiquinone oxidoreductase subunit V3	Q3U422	-1,091105268	0,467469
**SDHA**	succinate dehydrogenase complex flavoprotein subunit A	Q8K2B3	1,132224774	0,0577188
**SDHB**	succinate dehydrogenase complex iron sulfur subunit B	Q9CQA3	1,019306565	0,727502
**SDHC**	succinate dehydrogenase complex subunit C	F8WGB3	1,381577175	0,00263165
**SDHD**	succinate dehydrogenase complex subunit D	Q9CXV1	1,497884537	0,00199167
**UQCR10**	ubiquinol-cytochrome c reductase, complex III subunit X	Q5NCJ9	1,084144879	0,628202
**UQCRB**	ubiquinol-cytochrome c reductase binding protein	Q9CQB4	1,585364739	0,00320201
**UQCRC1**	ubiquinol-cytochrome c reductase core protein 1	Q9CZ13	1,103466495	0,0302956
**UQCRC2**	ubiquinol-cytochrome c reductase core protein 2	Q9DB77	1,307964858	0,0023692
**UQCRFS1**	ubiquinol-cytochrome c reductase, Rieske iron-sulfur polypeptide 1	Q9CR68	1,164235726	0,225653
**UQCRQ**	ubiquinol-cytochrome c reductase complex III subunit VII	Q9CQ69	1,421077458	0,0112891

Table S2: IPA analysis of the oxidative phosphorylation of female SAC mice. Blue color shows significantly downregulated proteins compared to WT mice.

Symbol	Entrez Gene Name	UniProt	Expression Fold Change	Expression p-value
**ATP5B**	ATP synthase F1 subunit beta	P56480	-1,0483	0,022013
**ATP5L**	ATP synthase membrane subunit g	Q9CPQ8	1,043214	0,37549
**ATP5O**	ATP synthase peripheral stalk subunit OSCP	Q9DB20	1,00571	0,653439
**COX5A**	cytochrome c oxidase subunit 5A	P12787	1,101418	0,49025
**COX7A2**	cytochrome c oxidase subunit 7A2	P48771	-1,04944	0,129319
**ND1**	NADH dehydrogenase, subunit 1 (complex I)	P03888	-1,48769	0,499383
**NDUFA10**	NADH:ubiquinone oxidoreductase subunit A10	Q99LC3	-1,09567	0,012843
**NDUFA13**	NADH:ubiquinone oxidoreductase subunit A13	Q9ERS2	-1,16658	0,049629
**NDUFA2**	NADH:ubiquinone oxidoreductase subunit A2	Q9CQ75	-1,10987	0,017984
**NDUFA3**	NADH:ubiquinone oxidoreductase subunit A3	Q9CQ91	-1,12179	0,00759
**NDUFA6**	NADH:ubiquinone oxidoreductase subunit A6	Q9CQZ5	-1,0644	0,048424
**NDUFA7**	NADH:ubiquinone oxidoreductase subunit A7	Q9Z1P6	-1,26195	0,017791
**NDUFA8**	NADH:ubiquinone oxidoreductase subunit A8	Q9DCJ5	-1,04613	0,052604
**NDUFB11**	NADH:ubiquinone oxidoreductase subunit B11	O09111	-1,06108	0,740741
**NDUFB3**	NADH:ubiquinone oxidoreductase subunit B3	Q9CQZ6	-1,0454	0,388971
**NDUFB4**	NADH:ubiquinone oxidoreductase subunit B4	Q9CQC7	1,053449	0,382914

**NDUFB7**	NADH:ubiquinone oxidoreductase subunit B7	Q9CR61	-1,16581	0,001242
**NDUFB9**	NADH:ubiquinone oxidoreductase subunit B9	Q9CQJ8	-1,07164	0,045184
**NDUFS1**	NADH:ubiquinone oxidoreductase core subunit S1	Q91VD9	-1,04716	0,041061
**NDUFS3**	NADH:ubiquinone oxidoreductase core subunit S3	Q9DCT2	-1,02622	0,054845
**NDUFS6**	NADH:ubiquinone oxidoreductase subunit S6	P52503	-1,12152	0,050392
**NDUFV2**	NADH:ubiquinone oxidoreductase core subunit V2	Q9D6J6	-1,23939	0,019085
**NDUFV3**	NADH:ubiquinone oxidoreductase subunit V3	Q3U422	-1,15421	0,059305
**SDHA**	succinate dehydrogenase complex flavoprotein subunit A	Q8K2B3	1,023128	0,346857
**SDHB**	succinate dehydrogenase complex iron sulfur subunit B	Q9CQA3	-1,1003	0,015305
**SDHD**	succinate dehydrogenase complex subunit D	Q9CXV1	-1,20444	0,147099
**UQCRC1**	ubiquinol-cytochrome c reductase core protein 1	Q9CZ13	-1,06154	0,008637
**UQCRC2**	ubiquinol-cytochrome c reductase core protein 2	Q9DB77	1,001478	0,120837
**UQCRFS1**	ubiquinol-cytochrome c reductase, Rieske iron-sulfur polypeptide 1	Q9CR68	-1,01547	0,61814
**UQCRQ**	ubiquinol-cytochrome c reductase complex III subunit VII	Q9CQ69	-1,0132	0,310037

Table S3: IPA analysis of the mTOR pathway in male SAC mice. Red colour shows significantly upregulated and blue colour significantly downregulated proteins compared to WT mice.

**Symbol**	**Entrez Gene Name**	**UniProt**	**Expression Fold Change**	**Expression p-value**
**Cdc42**	cell division cycle 42	P60766	1,540981	1,94E-05
**EIF3A**	eukaryotic translation initiation factor 3 subunit A	P23116	-1,2476	0,091012
**EIF3B**	eukaryotic translation initiation factor 3 subunit B	Q8JZQ9	-1,20117	0,022585
**EIF3C**	eukaryotic translation initiation factor 3 subunit C	Q8R1B4	-1,23968	0,120117
**EIF3D**	eukaryotic translation initiation factor 3 subunit D	O70194	1,09707	0,433826
**EIF3E**	eukaryotic translation initiation factor 3 subunit E	Q3UIG0	-1,07593	0,22065
**EIF3F**	eukaryotic translation initiation factor 3 subunit F	Q9DCH4	-1,28723	0,04318
**EIF3G**	eukaryotic translation initiation factor 3 subunit G	Q544H0	-1,46485	0,036465
**EIF3H**	eukaryotic translation initiation factor 3 subunit H	Q5M9L0	-1,64027	0,036758
**EIF3I**	eukaryotic translation initiation factor 3 subunit I	Q9QZD9	1,018038	0,877609
**EIF3J**	eukaryotic translation initiation factor 3 subunit J	Q3UGC7	-1,25137	0,03772
**EIF3L**	eukaryotic translation initiation factor 3 subunit L	Q8QZY1	-1,13806	0,456783
**EIF4A1**	Eukaryotic Translation Initiation Factor 4A1	Q5F2A7	-1,35793	0,00517
**EIF4A2**	Eukaryotic Translation Initiation Factor 4A2	P10630	1,005995	0,941128
**EIF4A3**	Eukaryotic Translation Initiation Factor 4A3	Q91VC3	1,040051	0,464287
**eIF4B**	eukaryotic translation initiation factor 4B	Q8BGD9	-1,06853	0,429906
**eIF4E**	eukaryotic translation initiation factor 4E	A0A0G2JH04	1,224622	0,193666
**EIF4G1**	Eukaryotic Translation Initiation Factor 4 Gamma 1	E9PVC6	1,009655	0,897905
**EIF4G2**	Eukaryotic Translation Initiation Factor 4 Gamma 2	F7CBP1	-1,04425	0,395205
**FKBP1**	Peptidyl-prolyl cis-trans isomerase	P26883	-1,09399	0,723345

**MAPK3**	mitogen-activated protein kinase 3	D3Z3G6	1,064703	0,777924
**PPP2CA**	Serine/threonine-protein phosphatase 2A catalytic subunit alpha isoform	P63330	-1,00567	0,968446
**PPP2R1A**	protein phosphatase 2 scaffold subunit Aalpha	Q76MZ3	1,05696	0,395291
**PTPA**	Serine/threonine-protein phosphatase 2A activator	Q543N6	-1,10334	0,387378
**RAC**	RAS-related C3 botulinum substrate 1	K7Q7T7	1,139008	0,371887
**RAP1A**	Ras-related protein Rap-1A	P62835	1,084862	0,315107
**RHOA**	Ras family member A	Q4VAE6	-1,00361	0,981248
**RHOT1**	Mitochondrial Rho GTPase 1	Q8BG51	1,199016	0,177205
**RPS10**	ribosomal protein S10	Q5M9K7	-1,10841	0,609755
**RPS11**	ribosomal protein S11	Q3UC02	-1,14374	0,300757
**RPS12**	ribosomal protein S12	Q6ZWZ6	-1,2036	0,1127
**RPS13**	ribosomal protein S13	Q5BLJ7	1,123551	0,474207
**RPS14**	ribosomal protein S14	P62264	-1,33818	0,018767
**RPS15**	ribosomal protein S15	D3YTQ9	-1,25502	0,142416
**RPS15A**	ribosomal protein S15A	Q5M9M4	1,011522	0,914649
**RPS16**	ribosomal protein S16	A4FUS1	-1,24287	0,009313
**RPS17**	ribosomal protein S17	Q5M9L7	-1,26688	0,101462
**RPS18**	ribosomal protein S18	Q561N5	-1,08507	0,329736
**RPS19**	ribosomal protein S19	Q9CZX8	-1,26948	0,021572
**RPS2**	ribosomal protein S2	Q58EU3	-1,11017	0,383971
**RPS20**	ribosomal protein S20	Q5BLK2	-1,41691	0,002367
**RPS21**	ribosomal protein S21	Q9CQR2	-1,43761	0,000159
**RPS23**	ribosomal protein S23	P62267	1,034375	0,777361
**RPS24**	ribosomal protein S24	P62849	-1,11807	0,41224
**RPS25**	ribosomal protein S25	Q58EA6	-1,0557	0,625492
**RPS26**	ribosomal protein S26	Q497N1	-1,60241	0,000878
**RPS27A**	ribosomal protein S27A	P62983	1,049891	0,212852
**RPS27L**	ribosomal protein S27L	Q6ZWY3	-1,22078	0,004931
**RPS28**	ribosomal protein S28	G3UYV7	-2,46397	0,007698
**RPS29**	ribosomal protein S29	P62274	-1,21994	0,417447
**RPS3**	ribosomal protein S3	Q5YLW3	-1,20102	0,133493
**RPS4Y1**	ribosomal protein S4Y1	Q545X8	-1,07143	0,501606
**RPS5**	ribosomal protein S5	D3YYM6	-1,17434	0,160589
**RPS6KA1**	Non-specific serine/threonine protein kinase	G3UZI3	1,25737	0,037294
**RPS7**	ribosomal protein S7	Q4FZE6	-1,61447	0,039958
**RPS8**	ribosomal protein S8	Q497E9	-1,26073	0,000361
**RPS9**	ribosomal protein S9	Q6ZWN5	-1,11871	0,262164
**RPSA**	ribosomal protein SA	P14206	-1,22301	0,014658
**RRAS2**	Ras-related protein R-Ras2	P62071	-1,08332	0,505504

87

Table S4: IPA analysis of the mTOR pathway in female SAC mice. Red colour shows significantly upregulated proteins compared to WT mice.

Symbol	Entrez Gene Name	UniProt	Expression Fold Change	Expression p-value
Cdc42	cell division cycle 42	P60766	1,165059	0,457934
EIF3A	eukaryotic translation initiation factor 3 subunit A	P23116	1,125472	0,007718
EIF3B	eukaryotic translation initiation factor 3 subunit B	Q8JZQ9	1,147002	0,013067
EIF3C	eukaryotic translation initiation factor 3 subunit C	Q8R1B4	1,297426	0,005495
EIF3D	eukaryotic translation initiation factor 3 subunit D	O70194	1,114022	0,163498
EIF3F	eukaryotic translation initiation factor 3 subunit F	Q9DCH4	1,226081	0,000242
EIF3I	eukaryotic translation initiation factor 3 subunit I	Q9QZD9	1,203921	0,009641
EIF3L	eukaryotic translation initiation factor 3 subunit L	Q8QZY1	1,289237	0,009817
eIF4B	eukaryotic translation initiation factor 4B	Q8BGD9	1,075572	0,123038
MAPK1	mitogen-activated protein kinase 1	P63085	1,01818	0,254812
PPP2R1A	protein phosphatase 2 scaffold subunit Aalpha	Q76MZ3	-1,07324	0,177206
RPS21	ribosomal protein S21	Q9CQR2	1,18445	0,000504
RPS23	ribosomal protein S23	P62267	1,18845	0,016624
RPS24	ribosomal protein S24	P62849	1,181007	0,157707
RPS29	ribosomal protein S29	P62274	1,182092	0,001764
RPSA	ribosomal protein SA	P14206	1,232653	0,001462
